# Comparison of Operational Jet Fuel and Noise Exposure for Flight Line Personnel at Japanese and United States Air Bases in Japan

**DOI:** 10.3390/toxics13020121

**Published:** 2025-02-05

**Authors:** David R. Mattie, Dirk Yamamoto, Kerrine LeGuin, Elizabeth McKenna, Daniel A. Williams, Alex Gubler, Patricia N. Hammer, Nobuhiro Ohrui, Satoshi Maruyama, Asao Kobayashi

**Affiliations:** 1 Air Force Research Laboratory, 711 Human Performance Wing (HPW), Wright-Patterson AFB, Greene County, OH 45433, USA; dirk.yamamoto.2@us.af.mil (D.Y.); kerrine.m.leguin.mil@health.mil (K.L.); elizabeth_anne.mckenna.2@us.af.mil (E.M.); daniel.williams.88@us.af.mil (D.A.W.); 2 374 MDG, Yokota AB, Nishitama 190-1211, Tokyo, Japan; alex.h.gubler.mil@health.mil; 3 39 MDOS/SGXW, Kadena AB, Nakagami 904-0112, Okinawa, Japan; dochammer@flyprescribewin.com; 4 Aeromedical Laboratory, Japan Air Self-Defense Force, Ichigaya 162-8804, Saitama, Japan; oruij3n@inet.aci.mod.go.jp (N.O.); maruyamaq0t@inet.aci.mod.go.jp (S.M.); 5 Air Development & Test Command HQ, Japan Air Self-Defense Force (Ret), Fuchu City 162-8801, Tokyo, Japan; asaokobayashi@jcom.home.ne.jp

**Keywords:** jet fuel, volatile organic chemicals, noise dosimetry, audiology, human exposure

## Abstract

Flight line personnel are constantly exposed to noise and jet fuel while working on flight lines. Studies suggest that jet fuel in combination with noise affects hearing loss more than noise exposure alone. This study examined the combined effects of jet fuel and noise exposure on the hearing of flight line personnel stationed at Japan Air Self-Defense Force Air Bases (Hamamatsu, Matsushima, Hyakuri, Yokota, and Iruma) and US Air Force Air Bases (Kadena and Misawa) in Japan. Samples were collected from all participants, 97 flightline-exposed and 71 control volunteers, to measure their individual noise levels with a personal sound level meter and volatile organic chemicals (VOCs) with a chemical sampling pump during a single shift. Blood samples were collected post shift. Urine samples (entire void) were collected prior to the shift (morning first void) and post shift. VOCs were measured in air, blood, and urine. An audiometric test battery, consisting of immittance measurements, audiograms, distortion product otoacoustic emissions, and the auditory brain response, was conducted after the shift to examine the hearing of participants. Total VOCs in personal air samples were in the ppb range for each group. Tinnitus and temporary hearing loss were reported in audiological histories but were also present in some controls. Noise levels on the flight line were greater than the action level for requiring hearing protection and exceeded exposure limits, but all exposed subjects reported wearing hearing protection. Audiometric tests identified significant differences and trends between flight line and control personnel, indicating the potential for hearing disorders. In spite of very low levels of VOC exposure and wearing hearing protection for noise, there is still the potential for hearing issues in flight line personnel.

## 1. Introduction

Personnel who work in and around aircraft are at risk of developing hearing loss. Recent studies suggested that jet fuel in combination with noise exposure can cause hearing loss more than noise alone [1,2,3]. Studies have shown that pilots, flight crews, aircraft technicians, and mechanics have high rates of hearing loss compared to unexposed populations from analyses of large epidemiological databases [4,5]. Aircraft technicians and mechanics in a Swedish commercial aviation company were found to have a higher rate of hearing loss at younger ages compared to an age-matched reference group, and subjective hearing loss was associated with self-reported exposure to solvents [6]. In addition, military fighter and helicopter pilots are known to be at a higher risk of developing hearing loss compared to commercial pilots [7]. Hearing loss typically takes on a high-frequency sloping configuration, even with the use of hearing protection such as earplugs, headsets, or helmets [5].

A retrospective cohort study was conducted by Williams et al. [8] using jet propulsion-8 (JP-8) and hearing test data from the Department of Defense on occupational and environmental health. Hearing tests were compared for personnel exposed to noise and those exposed to both noise and JP-8 from 1996 to 2013. Personnel aged 17–44 years who were exposed to JP-8 were more likely to have an occupational hearing shift than their non-exposed peers of the same age. When the mean threshold change was examined between the first and last audiograms of the personnel, a statistically significant difference was found at 3000 hertz (Hz) and 4000 Hz in the JP-8-exposed group. The results of this epidemiological study suggest a synergistic effect on hearing loss when workers are exposed to both noise and JP-8.

The United States Armed Services and North Atlantic Treaty Organization (NATO) have adopted JP-8 as their standard fuel [9,10]. The Japan Air Self-Defense Force (JASDF) has switched to jet fuel aviation 1 (Jet A1), which is JP-8 without additives. The US Air Force (USAF) now designates jet fuel aviation (Jet A) with the current AF performance additives as F-24. Jet A and Jet A1 are very similar in chemical composition, but Jet A1 has a lower freezing point. Jet A is mainly used in the US commercial aviation industry.

Preliminary epidemiological analyses of auditory records from aircraft maintenance personnel exposed to both noise and jet fuel (primarily the more volatile jet propulsion-4 (JP-4)) suggest that concurrent exposure may potentiate hearing loss at jet fuel concentrations below the currently established safe occupational exposure limits [11]. Potentiation of hearing loss was confirmed in experiments where rats were exposed to JP-8 and non-damaging noise levels [12]. Recent animal studies have revealed that exposure to JP-8 combined with noise may result in the loss of pre-neural cochlear sensitivity, which was shown by suppression of distortion product otoacoustic emissions (DPOAEs), increased auditory thresholds, and depletion of cochlear sensory cells, as evidenced by cytocochleograms, which plot the percentage of missing outer hair cells [9,12,13]. In the experimental animal models, the effect of combining jet fuel and noise exposure was greater than that of the individual exposure to jet fuel or noise. An additional study by Guthrie et al. [14] examined the same functional and structural assays of the presynaptic sensory cells, but added neurophysiologic studies of the auditory pathway, by conducting an auditory brain response (ABR) test. Results revealed that peripheral auditory function was not affected by individual exposure, and there was no effect when the forms of exposure were combined using this exposure paradigm and animal model. However, brainstem encoding of stimulus intensity measured by ABR was impaired. All of these findings are particularly relevant because military and government regulations regarding toxic exposure are often based on exposure to a single agent and less is known about combined exposure to jet fuel and noise.

A review of the literature by Warner, Fuente, and Hickson [15] examined the effects of combined exposure to JP-8 jet fuel (or its aromatic solvent components) and noise on the auditory nervous system in humans. Their search found 178 articles but only 6 met their inclusion criteria and were summarized in the review. One article in the review was by Kaufman et al. [11], where exposure was to JP-4 jet fuel only. The other five examined the effects of individual solvents or mixed solvents. The conclusions of the literature review suggest that there is an association between aromatic solvents and jet fuel, in combination with noise and central auditory dysfunction/hearing loss. The articles considered in this review indicate that the assessment of the auditory nervous system, including pure-tone audiometry, distortion product otoacoustic emission, auditory brainstem response, and behavioral functional assessments, should be conducted as part of a comprehensive test battery for military members exposed to both noise and solvents in the workplace. Based on current documented evidence, there is a need for a closer investigational paradigm, to survey and document the auditory and environmental exposure of flight line personnel via hearing decrement assessments.

A previous collaborative research effort between the JASDF and the USAF correlated exposure and biomarkers for JP-4 and compared them with those from JP-8 [16,17]. The JASDF measured jet fuel components in air, blood, and urine, while the USAF analyzed them for biomarkers. Personal exposure of 48 VOCs was measured in F-15 and C-130 flight line crews and non-exposed control subjects (mainly hospital workers). This study suggested that the level of exposure to jet fuel or jet fuel combustion products is dependent on the type of aircraft and fuel. Moreover, the trend of VOCs is that workers in operational environments are exposed through the lungs with entry into the blood, and then the VOCs are metabolized, resulting in metabolites in the urine. This study was designed to characterize JP-4 and JP-8 jet fuel components and their amounts in blood, urine, and the breathing zone.

In order to gain further knowledge regarding combined jet fuel and noise exposure and specific auditory health effects of flight line personnel, a direct human exposure response comparison study during military operations was performed. The purpose of this study was to examine the effects of combined exposure to jet fuel and noise on hearing in flight line personnel. This study also examined the characteristics of VOC exposure in the flight line environment for each aircraft and fuel type at the bases sampled.

## 2. Methods

This study was the result of a collaborative research effort between the JASDF and the USAF, who worked together as a combined team at air bases (ABs) in Japan to collect data. The JASDF then analyzed jet fuel components as fifteen VOCs in personal air samples from the flight line air environment, blood, and urine, while the USAF analyzed noise dosimetry and an audiometric test battery, consisting of immittance measurements, audiograms, DPOAE, and ABR.

### 2.1. Recruitment of Study Participants and Demographic Data

This study recruited flight line personnel participants from F-15 and F-16 USAF ABs exposed to JP-8 and individuals from T-4, F-2, and F-4 JASDF ABs exposed to Jet A1 or JP-4. Non-exposed control participants were mainly medical personnel sampled in an identical manner as flight line personnel. Controls were matched to the number of flight line personnel for JP-8 at USAF ABs and to the number of flight line personnel for JP-4/Jet A1 at JASDF ABs. Non-exposed control participants in the JASDF were recruited from Yokota and Iruma ABs because there are only a few medical personnel who could serve as non-exposed control participants at typical JASDF ABs such as Hamamatsu, Matsushima, and Hyakuri. Participants were volunteers who were active-duty (JASDF and USAF) flight line or medical personnel. Participants could be male or female but their age had to be under 35 years old to avoid changes in hearing due to aging.

Participants were asked about the following demographic information [18]: name, which was replaced with a base-specific number to de-identify participants; career field; rank; years of service; age; gender; hobbies (other fuel or solvent exposure, such as working on car or truck engines, either on or off duty); and last time they fueled a government or personal vehicle, with the type of fuel. At the end of the shift, questions were asked about exposure during the shift as well the work activities associated with jet fuel and jet exhaust, including time on the flight line (location of job, physical activities). Additional questions were about exposure to spills of any kind; any direct dermal exposure to jet fuel or other forms of exposure such as solvents and cleaning fluids; whether they smoked (if yes, number of cigarettes, cigars, heated tobacco products, and/or vapes smoked); and how much caffeine they consumed. They were also asked about personal protective equipment. A second post-shift questionnaire was for the audiological history. Questions included the incidence of temporary hearing loss, tinnitus, ear infections, dizziness, ear pain, family history, and neurological or chronic medical conditions. They were also asked for information about off-duty and recreational noise exposure as well as usage of any herbal supplements. There were one hundred and sixty eight individuals who voluntarily joined this study at five JASDF ABs and two USAF ABs in Japan (Table S1).

Participants were divided into eight groups by the Aeromedical Laboratory (AML) of the JASDF: CJ (non-exposed controls at the JASDF); CK (non-exposed controls at Kadena); CM (non-exposed controls at Misawa); T-4 (Jet A1-exposed T-4 flight line crews at Matsushima and Hamamatsu); F-2 (Jet A1-exposed F-2 flight line crews at Matsushima); F-4 (JP-4-exposed F-4 flight line crews at Hyakuri); F-15 (JP-8-exposed F-15 flight line crews at Kadena); and F-16 (JP-8-exposed F-16 flight line crews at Misawa).

### 2.2. Sampling of Noise, Blood, Urine, and Audiology

Samples for noise dosimetry, VOCs in the air, blood, and urine, and an audiometric test battery, consisting of immittance measurements, an audiogram, DPOAE, and ABR, were collected according to a typical sequence of events at each base. Prior to the shift, urine was collected, and a personal air sampler and noise dosimeter were placed on each subject. During a shift, each subject performed work duties as usual. Post shift, after flight line operations or work in a control setting, the personal air sampler and noise dosimeter were removed, blood was drawn, and the second urine sample was collected.

**Figure 1:**
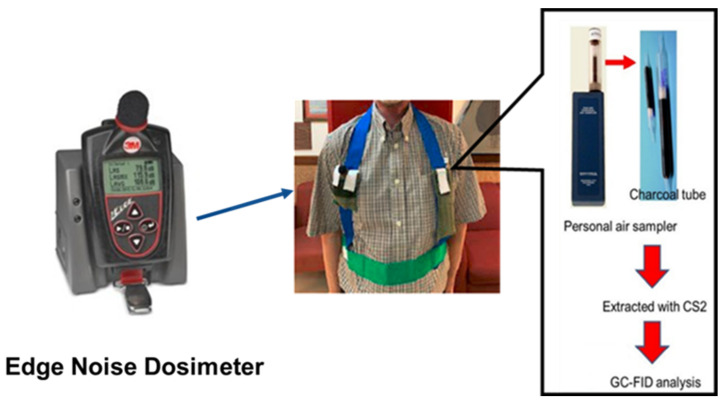
Personal air-sampling device, personal air monitor (PAS-500), and charcoal tube (ORBO32 small, SUPELCO) in the left pocket of the person. On the right is an analytical scheme for volatile organic chemicals (VOCs) (images are property of JASDF/AML co-authors). On the left, and in the right pocket of the person, is the Quest™ EG5, TSI Edge Noise Dosimeter (now discontinued; image of person is David Mattie, the first author, in a photo taken by a USAF co-author).

To measure noise levels during the shift, prior to the shift, each subject had a Quest™ Edge Noise Dosimeter (Model EG5, TSI, Oconomowoc, WI, USA) personal sound level monitor placed in the right pocket of a belt they wore with shoulder straps, designed by the JASDF AML (Figure 1). Values assigned for the noise dosimeter during sampling are shown in Table 1. The criterion level is the OSHA action level for noise exposure. The exchange rate is the decibel level increase for which the exposure time needs to be cut in half. The integrating threshold is the A-weighted sound pressure level at which a noise dosimeter begins to integrate the noise into the measured exposure. For USAF purposes, this is 80 decibels A weighting (dBA). Internal Threshold Enable equals True turns the integration on. Peak weighting can be A, C, or Z. A-weighting cuts off the lower and higher frequencies that the average person cannot hear, while C-weighting is for high noise or impulse noise exposure. The dosimeter was removed and turned off at the end of a shift. In addition to the sound levels, the meter also measured the length of a shift.

A personal air monitor (PAS-500, Spectrex, Redwood City, CA, USA) was placed into the left pocket of a vest worn by the study participant (Figure 1). The monitor went into the pocket with just the tip of the charcoal tube (ORBO32 small, SUPELCO, Bellefonte, PA, USA) extending from the pocket. Prior to the analysis, the charcoal was removed from the tube. VOCs as well as higher chain compounds (up to C15 and C16) were extracted from the charcoal using carbon disulfide. The sample extracts were analyzed using a gas chromatograph with a flame ionization detector (Gas Chromatography-Flame Ionization Detector (GC-FID), Shimadzu Corporation, Kyoto, Japan) for hydrocarbons, with boiling point 36–216 °C, and aromatic hydrocarbons according to the standard NIOSH Methods: 1500, Issue 3 [19] and 1501, Issue 3 [20], respectively.

Each participant had their blood drawn at the clinic, base hospital, or an approved designated area for that base. Blood was drawn into a 5 mL blood collection tube for chemical analysis with heparin. The whole-blood samples were stored chilled (4 °C) in the dark until analysis. For each subject, a 3 mL of portion of whole blood was transferred to a 15 mL SPME (solid phase microextraction) headspace vial. Samples were spiked with 20 μL of an internal standard mixture. Samples were analyzed by a gas chromatography–mass spectrometer (GC/MS) detector in electron ionization (EI) mode using a Shimadzu Corporation GC/MS TQ8040-NX (Kyoto, Japan).

Urine samples (entire void) were collected prior to a shift (first void in the early morning) and post shift to determine the urine levels of jet fuel components. The urine weight was recorded, and the urine samples for chemical analysis were stored chilled (4 °C) in the dark until analysis by AML for jet fuel components. To permit analysis, the urine was transferred to a solid-phase microextraction (SPME) headspace vial. The sample was spiked with an internal standard mixture. Samples were analyzed for jet fuel components by GC/MS in EI mode using a Shimadzu GC/MS TQ8040-NX and reported per 100 µg of creatinine (Cr).

The 15 VOCs measured in personal air samples, blood, and urine included 10 straight-chain alkanes: n-hexane, n-heptane, n-octane, n-nonane, n-decane, n-undecane, n-dodecane, n-tridecane, n-tetradecane, and n-pentadecane, and 5 aromatic hydrocarbons: benzene, toluene, ethylbenzene, m,p-xylene, and o-xylene. A technical report was published for the VOC analysis by Orui et al. [21]. The detection limits for each VOC in ng/mL, which is also ppb, are as follows: hexane 29.6; benzene 30.1; heptane 30.3; toluene 30.8; octane 30.2; ethylbenzne 30.6; m-xylene and p-xylene 30.6; o-xylene 30.3; nonane 29.5; decane 29.4; undecane 30.6; dodecane 30.6; tridecane 30.3; tetradecane 29.5; and pentadecane 29.4.

### 2.3. Audiological Tests

Audiometric threshold measurements were conducted post shift in an approved, existing sound booth or using a portable Defense Occupational and Environmental Health Readiness System-Hearing Conservation (DOEHRS-HC) audiometer in a room with a closed door and minimal noise interference. At Misawa AB, a GSI Audio Star Pro audiometer (Grason-Stadler, Eden Prairie, MN, USA) was used in a sound booth. At Iruma AB, a Model AA-77A audiometer (RION Co., Ltd., Tokyo, Japan) was used in a sound booth. The following battery of tests were conducted for each subject. Otoscopy: The ear canals were examined prior to audiologic measurements to confirm they were free from occlusion (cerumen) and presented a normal tympanic membrane. Ear cleaning was performed as required. A Welch Allyn diagnostic otoscope was used to examine the ears. For immittance, the status of the middle ear was evaluated by routine tympanometry and acoustic reflex testing (immittance audiometry) using the Interacoustics Titan Clinical Module (Middelfart, Denmark). Acoustic reflex thresholds were tested at 500, 1000, and 2000 Hz, both ipsilaterally and contralaterally, with a deflection criteria of 0.03 mL. Acoustic reflex thresholds were considered to be ipsilateral unless identified as contralateral or contra. The DPOAE measurements were collected in both ears separately from 2000 to 8275 Hz using the appropriate diagnostic signal -o-noise ratios for response calculations with the Interacoustics Titan Clinical Module. The ABR measurements were made with a two-channel electrophysiologic system using far-field electrode recordings (averaging), a SOAP-KALMAN Weighted Algorithm, and insert-earphones for stimulus delivery. The following electrode array was used for each test subject: surface electrodes were placed with the non-inverting electrode at Channel 1 active-Cz (high forehead or vertex), the inverting electrodes at A1 and A2 ear lobes for the left and right ears, respectively, and the ground electrode on the lower forehead. Electrodes’ external and internal (subject) noise interference was minimized by placing the equipment away from light and electrical inputs and by supine positioning the subjects. Two conditions (fast rate and slow rate) were measured to obtain findings in each ear for every test subject, with each condition using a rarefaction polarity. The first stimulus was an 80 dB sound pressure level (SPL) air-conducted click with a 27.5/second stimulus rate, and the second used an 80 dB SPL air-conducted click with an 85.9/second stimulus rate. Recordings were collected at least two times in each condition with a minimum of 1000 sweeps for replicability using the Vivosonic Integrity V500 8.4.1 Auditory Brainstem Response system (Toronto, ON, Canada). The I, III, and V waves’ latencies and I and V waves’ amplitudes were manually identified by audiologist observation for the slow-rate stimulus. Wave V latencies and amplitudes were manually marked by audiologist observation for the fast-rate stimulus. To obtain audiograms, audiometric thresholds were measured with TDH (total dynamic head) headphones at 500, 1000, 2000, 3000, 4000, and 6000 Hz in 5 dB steps in each ear using a modified Hughson–Westlake behavior testing method. Testing was completed using DOEHRS-HC software and hardware.

### 2.4. Statistical Analysis

The comparison of the mean age, working experience years, and concentrations of total VOCs in the personal air samples, blood, and urine among each group was completed using Mann–Whitney U-tests with Bonferroni correction. A *p*-value < 0.0018 (0.05/28) was considered a statistically significant difference. The comparison of total VOCs in urine prior to the shift and post shift, in the smokers and the non-smokers, was completed using the Wilcoxon signed-rank test. A *p*-value < 0.008 (0.05/6) was considered a statistically significant difference. All analyses were conducted in SPSS (version 24, SPSS Inc., Chicago, IL, USA).

The comparison of means for audiometric data was performed by *t*-tests with a *p*-value accepted as less than 0.05. Regression analysis was then conducted because of the inability to collect data from subjects only exposed to noise or only exposed to jet fuel in this study. The audiometric endpoints that were significantly different between flight line participants and controls were chosen for regression analysis. There were 28 statistically significant endpoints combined for ABR, DPOAE, and Immittance. For each significant endpoint, a regression utilizing the Analysis ToolPak in Excel was conducted for noise and then VOCs. First, the data points for the audiometric tests that were significant were tested against the corresponding noise data points for the aircraft involved, and the degree of association and significance recorded. Data points were the participants’ individual average noise exposure level measured by the noise dosimeter they wore versus their audiometric test values. Then, the audiometric data points were tested against their total VOC level, measured in the air by the personal air monitor sampling pump they wore at their breathing level. The air data were used because there were fewer non-detection results than in the blood. Fifty-six regressions were conducted and summarized based on the degree of association and *p*-value. The correlation coefficient (r) is a measure of the closeness of association of the points in a scatter plot to a linear regression line based on the points being compared. A perfect positive association has an r value of +1.0. A very strong association is between +0.8 and 1.0, a strong association is +0.6 to 0.8, a moderate association is +0.4 to 0.6, a weak association is +0.2 to 0.4, and a very weak or no association is 0.0 to +0.2 [22].

## 3. Results

### 3.1. Demographic Data, Personal Protective Equipment, and Exposure Summary

The mean age of the F-15 group was significantly lower than for CJ, CK, CM, and T-4 (Table 2). The mean age of the F-16 group was also significantly lower than for CK and CM. No significant differences were found among the other groups. The working experience (years in military service) of the F-15 group was significantly lower than those of the other groups, except for CJ and F-16. The average working experience, when regarded as years in their career field, was less than the time in military service, although for Kadena and Misawa, these two results were closer than in the other groups. The smoking rates were higher in the jet-fuel-exposed groups (T-4, F-2, F-4, and F-15: 35.0–71.4%), with the exception of the F-16 group (16.7%), than in the control groups CJ, CK, and CM (22.7–26.7%). The proportion of female participants was higher in the control groups (20.6–50.0%) than in the jet-fuel-exposed groups (0–16.7%).

Demographic data for age, experience (years in service and time in career field), gender, hobbies involving VOCs, tobacco use, and caffeine intake for the JASDF Air Bases can be found in Table S2 and for USAF Air Bases in Table S3.

Questions that dealt with personal protective equipment and its use were also part of the post-shift questionnaire. Everyone working on the flight line wore hearing protection, either ear plugs, earmuffs, or both. Most USAF personnel wore both, while more JASDF personnel wore only earmuffs. More JASDF personnel briefly removed their hearing protection than USAF personnel, with the times in minutes for JASDF and only in seconds for USAF personnel. The most common noises heard by flight line personnel were from jet engines and aircraft ground equipment (AGE). Noise exposure was continuous when either was running, but exposure varied throughout the shift. No one reported an impulse noise. All JASDF participants reported that the noise exposure was typical for their shift, while for the USAF, all Kadena AB and 10 out of 18 Misawa AB personnel said the noise exposure on the day they were sampled was typical for their shift.

The questions that corresponded to the number of instances of exposure on the flight line were subjective and qualitative (approximate), so they were summarized for the entire study. The time spent around refueling by the participants in this study was an approximate average of 15 min for all bases. The average time around running engines was 3 h. The average time on the flight line was between 3.5 and 4 h. Out of 97 exposed participants, all but 6 reported the smell of exhaust and all but 10 reported the smell of jet fuel. Exposure to spills involved jet fuel, hydraulic fluid, or engine oil. Exposure other than jet fuel was to hydraulic fluid, engine oil, isopropanol, and for F-16 maintainers, hydrazine. Dermal exposure to jet fuel or aircraft fluids occurred but on a more limited basis of 30% of flight line participants. The average level of physical activity during a shift was moderate.

### 3.2. VOCs in Personal Air Samples

The frequencies of detection and the mean concentrations of the components of VOCs in the personal air samples are shown in Table 3. The flight line personnel for the F-4 and F-15 were exposed to relatively higher levels of chemicals more frequently, as highlighted for cells that show the frequencies of detection of VOCs to be > 60% and the mean concentrations of VOCs to be > 1.0 ppb in the personal air samples. Differences in the components of VOCs in the personal air samples were observed between each jet fuel exposure group. The differences in the components of VOCs in the air samples between JP-4, JetA1, and JP-8 in each type of aircraft were considered as corresponding to each fuel’s composition. JP-4 primarily consists of C4 to C16 hydrocarbons, and JP-8 and JetA1 primarily consist of C9 to C16 hydrocarbons. In addition, JP-8, which is in the kerosene class, has less benzene and n-hexane than JP-4, which is a wider cut of aromatics.

The toxicity of two or more substances is assumed to be additive [23]. Exposure exceeding the permissible exposure limits is considered to have occurred if the value calculated by the ACGIH [23] additive mixture formula exceeds 1. The total VOCs in the personal air samples in each group were extremely low compared to recommended permissible exposure limits in Japan and the United States for both individual chemicals (Table S4) as well as for the additive formula proposed limits (Table S5). Such trace amounts of VOCs are also known to occur in carpet and wallpaper adhesives and can be detected in office and home environments.

### 3.3. VOCs in Blood

The frequencies of detection and the concentrations of VOCs in the blood of participants are shown in Table 4. Results with frequencies of detection of VOCs > 60% and concentrations of VOCs > 0.1 ng/mL in blood are highlighted in the table. Toluene was detected in all groups. Consistent with breathing zone air samples, differences in the VOC components in the blood were observed between the jet fuel exposure groups. The differences may be due to differences in the composition of the respective jet fuels and the performance of the respective jet engines. The highest frequency of detection and higher concentrations of VOCs in the blood were found in flight line workers exposed to JP-4. The bases with JP-8 had the next-highest values for frequency and concentrations. On the other hand, some components of VOCs in the blood were also found at a high frequency of detection and with concentrations similar to or lower than in the control groups at Kadena and Misawa ABs. Toluene was found at 100% in all controls, with a concentration higher than T-4 but less than F-2, F-4, F-15, and F-16. It is possible that these control levels do not reflect VOCs in the work environment.

### 3.4. VOCs in Urine: Pre-Shift and Post-Shift Summary

A frequency of detection of a VOC at > 60% and a concentration > 0.1 ng/100 µg Cr in the urine prior to a shift were only seen for benzene in F-4 [21]. Meanwhile, a frequency of detection of a VOC at > 60% and a concentration > 0.1 ng/100 µg Cr in the urine after the shift were only seen for benzene in the F-4 group and toluene in the F-15 group. Benzene had a high concentration in the F-15 group post shift, but the frequency was only 30.4%. Benzene was not found in high concentrations for air and blood in the F-15 group. Benzene was found at high frequencies and concentrations in the personal air samples, blood, and urine prior to the shift and in the urine post shift for the F-4 group. The volume of urine post shift required for analysis could not be collected for two participants in the F-4 group. Overall, total VOC concentrations in urine samples were significantly higher post shift than prior to the shift in Japanese controls and the T-4 group, but not significantly different in the other groups.

### 3.5. Comparison of VOCs in Blood and Urine in Smokers and Non-Smokers

The total VOCs were compared in the personal air samples, blood, and urine prior to the shift and the urine post shift in smokers and non-smokers (Figure S1). Participants were divided into four groups: NC, non-smoker control participants; SC, smoker control participants; NE, non-smoker jet-fuel-exposed participants; and SE, smoker jet-fuel-exposed participants. The VOCs in the urine prior to the shift and post shift for the NE and SE groups were significantly higher than in NC. On the other hand, no significant differences were found between NC and SC and between NE and SE for total VOCs in the personal air samples, blood, and urine prior to the shift and post shift. No association was found when the relationship was examined between VOCs and the questionnaire survey items for years of service, age, gender, and hobbies.

### 3.6. Sound Level Measurements

Noise dosimetry is reported for the JASDF and USAF air bases in Table 5. The Hyakuri AB flight line was the loudest for the JASDF, as they were still flying F-4 fighters at the time of the sampling. The loudest aircraft were the F-15s flown at Kadena AB. The USAF uses OSHA’s action level of 85 dB and NIOSH’s 3 dB exchange rate in its calculations (see Table 1). Z-weighting was chosen for peak weighting because it is a linear response range that represents both A- and C-weightings for peak noise levels. Projection time is the assumed time of the shift. Since the shift time was projected to be 8 h, a slow response time was appropriate. These values are used to evaluate the noise exposure and calculate an actual time-weighted average for the time of sampling. The dosimeter had two meters, and one was set as an A-weighting frequency filter while the other was set to C-weighting. The two meters cover the normal hearing range (A) and can better measure loud noises greater than 100 dB (C).

### 3.7. Audiological History

The audiological histories are summarized for JASDF air bases in Table S6 and for USAF air bases in Table S7. Tinnitus was only reported for the F-4 flight line personnel for JASDF air bases, but was also reported by one control participant at Iruma AB who had rare incidences of ear ringing. For USAF air bases, tinnitus was reported in both exposed and control participants, but there were more exposed (5 of 23 Kadena; 10 of 18 Misawa) than control (2 of 22; 5 of 15, respectively) participants. Many history items were reported for controls at both JASDF and USAF air bases. Since audiological histories are subjective in nature and consisted of limited numbers of responses (data points), they were only summarized by means, standard error of the mean, and number of participants, with no statistical analysis.

### 3.8. Audiological Tests: JASDF Air Bases

In the tables, significant differences are shown as *p*-values in bolded dark red. If a *p*-value was 0.20 or less, it was bolded in black to indicate a potential trend. Data are available in the Supplemental File. Immittance data are measured as static compliance (SC) in cubic centimeters (cc); peak pressure (P) in decapascals (daPa); equivalent ear canal volume (ECV) in cc; and acoustic reflex (AR), which was the ipsilateral and contralateral (C) AR in dB.

For the T-4 aircraft flight line personnel, only static compliance was significantly increased in the immittance test (Table S8) for the right ear (0.75 ± 0.07 vs. 0.58 ± 0.04; *p* < 0.03). Left ears showed a trend towards significance (0.82 ± 0.09 vs. 0.67 ± 0.05; *p* = 0.14); however, these differences were more statistical artifacts than an indication of potential hearing loss. Also trending in the left ears was the peak pressure (*p* = 0.10), which was normal for the control (−8.0 ± 2.08) and T-4 (−3.54 ± 1.54) participants; and the acoustic reflex threshold at the contralateral 2000 (92.7 ± 1.2 vs. 95.2 ± 0.9; *p* = 0.11), which showed a true trend towards hearing loss.

For DPOAE, only 5666 Hz was significantly decreased in the right ears (30.3 ± 1.3 vs. 26.8 ± 11; *p* = 0.048) of flight personnel, while 2920 Hz was significantly decreased (23.3 ± 1.0 vs. 19.9 ± 1.0; *p* = 0.02) in the left ears (Table S9). There were three trending decreases in amplitudes for T-4 participants but no consistent differences across frequencies (2656, 3210, 3529 Hz) and ears.

For slow-rate ABR, shown in the upper portion of Table 6, the Wave I to Wave V latency was significantly decreased in the right ears but not in the left. However, the Wave I amplitude was significantly decreased in the left ears, as was the amplitude-to-latency ratio for Wave 1. For fast-rate ABR, with the results given in the lower portion of Table 6, Wave V latency was slightly slower in the right ears than the left in controls but was not different between T-4 participants and controls. Otherwise, there were no differences between the right and left ears for control or T-4 participants. There was a strong trend for a greater fast-rate Wave V latency in the left ears of T-4 participants than controls, but the Wave V amplitudes and amplitude-to-latency ratios were not different. Meanwhile, in the right ears, the trend was the opposite, with the Wave V amplitudes and amplitude-to-latency ratios showing a slight trend of differences between controls and T-4 participants and no difference for Wave V fast-rate latency. Overall, there was a trend toward hearing issues for flight line personnel working on T-4 aircraft.

For the F-2 flight line study participants, only the ipsilateral acoustic reflex threshold at 1000 Hz was significantly decreased in the immittance test (Table S10) for the left ear (87.0 ± 0.9 vs. 82.5 ± 1.58; *p* = 0.039). There was a trend of a decrease in right ears at 500 Hz (95.2 ± 1.1 vs. 91.0 ± 3.3; *p* = 0.16), but there was no trend towards significance in the left ears at 500 HZ or at the other frequencies, other than 1000 Hz. However, the differences showed the opposite effect, with the exposed ears showing a better function, so this could represent a statistical artifact.

For DPOAE, there were multiple significant decreases for amplitudes in both the right and left ears, indicating functional changes in the cochlea (Table 7). Not all differences were seen in both ears, although there were trends in the left ear when the right ear average was significantly decreased. At 5154, 5666, and 6229 Hz, there were decreases in the left ear not seen in the right ear, although only 5666 Hz was significantly decreased in the left ear. This was the test with the most significant hearing changes. However, given the small sample size of only seven exposed participants, our results need to be interpreted cautiously.

There were no significant differences and we found only one slight trend in the Wave I amplitude-to-latency left ear ratio for ABR results in F-2 flight line personnel (Table S11). Overall, there was a trend toward hearing issues for flight line personnel working on F-2 aircraft.

Audiology test results for F-4 flight line participants only showed a trend for elevation in the immittance test for the ipsilateral acoustic reflex threshold at 2000 Hz (88.3 ± 1.1 vs. 91.0 ± 1.56; *p* = 0.16) in the right ears (Table S12). There were no significant differences for the means of any frequencies for either ear.

For DPOAE data at Hyakuri AB, there were no significant differences in either the right or left ear means (Table S13). There were only two trends in the left ear, one for the distortion product response at 2920 Hz (23.3 ± 1.05 vs. 20.3 ± 1.28; *p* = 0.085) and one at 3529 Hz (25.06 ± 1.03 vs. 22.49 ± 1.53; *p* = 0.16). The F-4 data were in contrast to the T-4 and F-2 data, which showed significant hearing changes for this test.

Although there were no significant differences for the ABR endpoints in F-4 flight line personnel, there were six results that showed a trend for differences between exposed and control participants (Table 8). Wave V showed a trend in both ears. Furthermore, the Wave I amplitude showed a trend in both ears, as did the amplitude/latency ratio for Wave I. Overall, there was a trend toward hearing issues for flight line personnel working on F-4 aircraft.

Audiogram data for JASDF T-4 flight line participants are shown in Table 9. Hearing thresholds up to and equal to a 20 dB hearing level (HL) are considered normal. Values 26 to 40 are considered mild hearing loss. At 500 Hz, it is much easier for background noises to impact the results. Increases at 6000 Hz are an indicator of hearing loss. Therefore, the audiogram data averages appear normal for the JASDF T-4 participants. However, when we considered individual participants, 5 out of 29 T-4 participants had a threshold greater than 25 dB in the right ear: 2 at both 500 and 6000 Hz, 1 at 4000 and 6000 Hz, 1 at just 500 Hz, and 1 at just 6000 Hz (with 25 dB at 500 Hz). An additional three participants had 25 dB at 6000 Hz, with one of them at 500 Hz as well. Eight more participants had thresholds of 25 dB at 500 Hz. Even though the average was normal for all thresholds, a total of 16 out of 29 T-4 participants had a value at 25 dB or greater at 500 and/or 6000 Hz. Except for the one participant with a 30 dB value at 4000 Hz, all of the mid-range thresholds (1000 to 4000 Hz) for the right ears ranged from −10 to 20 dB.

For the left ear, three of the participants with thresholds greater than 25 dB in the right ears at 6000 Hz also had thresholds greater than 25 dB at 6000 Hz, with only one of them also having a 35 dB value at 500 Hz. An additional subject had a threshold of 45 dB at 6000 Hz in the left ear but was normal at the other frequencies. An additional five subjects had a value of 25 dB at either 500 or 6000 Hz (only one at both). All the mid-range thresholds (1000 to 4000 Hz) for the left ears ranged from −5 to 20 dB. In the results for the right and left ears of T-4 subjects, many were at the high end of normal, with six showing mild hearing loss at some frequencies.

Audiogram data for JASDF F-2 flight line participants are shown in Table 10. The audiogram data averages appear normal for the JASDF F-2 subjects. For individual subjects, only one subject had a value of 30 dB at 6000 Hz in the right ear, with 25 dB at 500 Hz. Another participant had a value of 30 dB at 6000 Hz in the left ear, with 15 dB at 500 Hz. However, six out of seven subjects had a value of 25 dB or greater at either 500 or 6000 Hz. All of the mid-range thresholds (1000 to 4000 Hz) for the right and left ears ranged from −5 to 20 dB. The results for the right and left ears of F-2 subjects indicated four of them were at the high end of normal at one frequency (500 or 6000 Hz), with two showing mild hearing loss at 6000 Hz. Only one subject had a normal range of threshold values for both ears, at 0 to 15 dB across all Hz.

### 3.9. Audiologic Tests: USAF Air Bases

Audiograms did not show any significant differences between control and flight line participants (Table S14). There was a trend towards audiometric threshold levels being higher in exposed personnel at 1000 Hz for both the right (10.0 ± 0.8 vs. 8.0 ± 1.1; *p* = 0.14) and left (8.6 ± 1.4 vs. 5.3 ± 1.2; *p* = 0.09) ears. There was a less pronounced trend for the right ears only at 4000 Hz, in which those exposed had a better threshold than the controls (5.8 ± 1.4 vs. 9.3 ± 2.2; *p* = 0.18). Only one participant had a threshold of 35 dB for the left ear at 6000 Hz. The other frequencies for this subject were either 20 or 25 dB. Other than this one subject, the thresholds ranged from −5 to 20 dB for the right ear. Another subject had 25 dB at 3000 Hz for the left ear; otherwise, the range was −5 to 20 dB for the other 16 F-16 subjects. Only one subject showed mild hearing loss based on the audiogram data.

Acoustic reflexes in the immittance tests at 500 and 1000 contralateral thresholds were significantly increased for the right and left ears, but only at 1000 Hz for the right ear (Table 11). A peak pressure difference was seen in the right ear but only showed a trend in the left ear. However, both sets of peak pressures were within the normal range. There was a slight trend towards significance in the left ear at acoustic reflexes of 500 and 2000 Hz contralaterally in the right ear. However, at 2000 Hz contralaterally, there was a significant increase in the left ear.

For DPOAE, the only trend towards a decrease (Table S15) was for 6847 Hz in left ears (29.8 ± 1.8 vs. 26.7 ± 1.4; *p* = 0.18). This contrasts with the multiple significant F-2 results and is still less than the smaller number of changes seen for T-4 maintainers.

For the ABR slow-rate results in the upper portion of Table 12, only Wave I to III latency (significance in right ears; trend in the left) and Wave V amplitude (trends in both ears but almost significant in the left) had decreases in both ears. There were trends in the left ear only for latency for Waves I to V and the amplitude–latency ratio for Wave V (almost significant). There were significant differences in the right ear for Wave III latency (decrease) and Waves III to V latency (increase), with no trend or significance for the left ear. However, the Wave III latency and Wave I–III latency showed a better performance in the exposed ears. The Wave III–V latency in the right ear was the only significant difference, showing a decrease in performance. Although the exposed left ears had a higher Wave III–V latency, there was no trend to support the significance in the right ears. Wave III amplitudes were not recorded, so no Wave III amplitude-to-latency ratios could be determined, which may have provided more insights.

For the fast-rate results for Wave V, shown in the lower portion of Table 12, there were no differences between the right and left ears for latencies or the amplitude/latency ratios. However, there was a significant decrease in amplitude in the right ears of F-16 subjects and a trend of significance in the left ears. This was supported by strong trends in the amplitude/latency ratios for both the right and left ears.

The significant differences and trends for ABR in F-16 flight line personnel were in contrast to the results for F-2 flight line personnel, which only showed one slight trend. Meanwhile, they were comparable to the results for T-4 subjects, where there were the same number of changes. Overall, there was a trend toward hearing issues for flight line personnel working on F-16 aircraft.

The test results are shown for Kadena AB F-15 flight line participants in Table 13 (ABR slow rate and ABR Wave V fast rate). There were no significant differences for slow-rate latencies between flight line and control participants. However, there was a significant increase in the Wave V latency measure by the fast rate for the right ears (with no difference in the left ears). There were a number of trends of differences between control and flight line personnel. There was a slight trend of an increase in Wave I latency in the right ears, but no difference in the left ears. This supported the fast-rate Wave V latencies. In the left ears, there were three stronger trends for differences between control and exposed subjects, but the Wave I and V amplitudes and their ratio were higher in flight line subjects, which was the opposite of the expected impact on the auditory pathway. This trend was also seen in the fast-rate Wave V amps. However, the right ear latency data for the Wave I slow rate and Wave V fast rate indicated a possible trend toward hearing issues for flight line personnel working on F-15 aircraft based on the ABR tests.

### 3.10. Regression Analysis

Conducting a regression for each significant audiometric endpoint versus noise and VOCs resulted in a total of 56 regressions, which are summarized in Table 14. There were 28 regressions for DPOAE, 16 for ABR, and 12 for immittance. There were only two very strong associations where r, the correlation coefficient, was greater than 0.8. An r of 1.0 is a perfect positive correlation. There were 9 strong associations where r was between 0.6 and 0.8; 6 moderate associations with an r between 0.4 and 0.6; 15 weak with an r between 0.2 and 0.4; 10 very weak with an r between 0.0 and 0.2; and 14 with no apparent association because r was less than 0.01. There were only five significant regressions. Both of the very strong associations were DPOAE endpoints measured in the right ear at 2656 and 3529 Hz, and these represented two of the five significant regressions at *p* < 0.05. There was one strong regression for noise at 4265 Hz in the DPOAE test of the right ear, which was significant. For noise, there was one moderate association that was significant, for the ABR endpoint of Wave I–III latency in the right ear at a slow rate. The fifth significant endpoint was a strong association for VOCs in the left ear of the DPOAE test at 2656 Hz. However, the opposite ear for each of the significant regressions had either a weak or very weak association. Of the remaining strong associations that were not significant, two had *p*-values equal to 0.06 and two had *p*-values equal to 0.07, highly suggesting trends towards significance for additional DPOAE Hz values. There was one moderate association for Wave V latency, the right ear, a fast rate, and VOCs, which had a *p*-value of 0.06, suggesting a trend of an impact resulting from the exposure. The other four moderate associations had *p*-values in the range of 0.2 to 0.24, just outside of suggesting a trend. Of the 17 regressions that were moderate or high, 11 were for noise and 6 were for VOCs. Only four times did both noise and VOCs have associations that were moderate or high in the same ear at the same DPOAE Hz. For 4265 Hz in the right ear, noise was significant and had a strong association, while VOCs had a strong association but not a significant one. The left ear at 4265 Hz had a strong association for noise and was moderate for VOCs, with neither being significant. In the left ears, 2920 Hz produced a strong association for noise and moderate for VOCs, while at 5666 Hz, noise showed a moderate association and VOCs a strong one.

**Figure 2:**
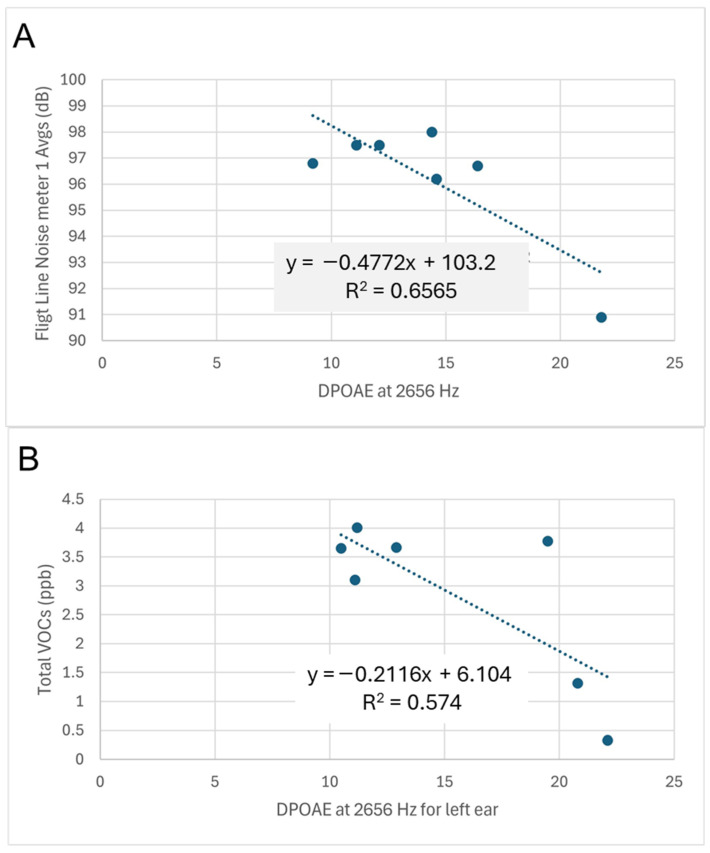
DPOAE values at 2656 Hz for seven participants who work on F-2 aircraft. (**A**) Versus right ear for noise with r = 0.81 and *p* = 0.03. (**B**) Versus left ear for total VOCs with r = 0.76 and *p* = 0.048.

The r-squared (r^2^ or R^2^) value or coefficient of determination indicates how well a regression fits the data. The highest r-squared values were for the two very strong associations, but were only 0.66 and 0.71 (Table 14). Between the type of data being analyzed and the low number of participants per aircraft, the r-squared values were low, in spite of higher associations and significance or near significance. As examples of the regression data, scatter plots are shown in Figure 2 for DPOAE at 2656 Hz for the right and left ears, which were both significant. The right ears had a very strong association for noise, while the left ears had a strong association for VOCs. The r-squared values were only 0.66 and 0.57, respectively. Of interest is that the aircraft with the most associations, greatest significance, and highest r-squared values had the lowest number of participants sampled, which was only seven.

## 4. Discussion

### 4.1. Participants

An ideal study design would involve participants exposed to noise alone and jet fuel alone. However, it is extremely difficult to find personnel on an operational air base who are only exposed to noise or just jet fuel. In addition, since a full week was required to sample sufficient participants in a group, the sampling teams would have required 5 weeks away on travel to complete data collection for four groups at each base. Plus, half a week at the beginning and a half a week at the end were required at each base to allow for travel and logistical issues. Therefore, the optimal study design was determined to involve control participants with no jet fuel exposure plus no flight line noise, and flight line personnel exposed to both jet fuel and noise. To address the lack of noise-only or jet-fuel-only participants, regression analysis was conducted for the significant audiometric endpoints.

Flight line personnel sampled were in their twenties, with USAF personnel younger than JASDF personnel on average. Controls tended to be older as there were a higher number of more senior personnel who were available to volunteer. However, controls were not that much older, so age-related hearing loss was not an issue. There were more female controls because of the higher number of females working in medical career fields. Also, the number of women working in maintenance flight line positions is much lower than that of men.

Overall, the numbers and types of participants suggest an impact of a combined exposure to noise and jet fuel on flight lines. The controls were sufficient to provide a group for comparison. An ideal comparison would involve each exposed participant being compared to a baseline set of data collected when they first started their careers. Unfortunately, audiometric testing as conducted in this study has not been performed at the start of careers.

Blood and urine were collected for VOC determination, with blood also used to measure miRNA, while urine was used to measure metabolites. Both the miRNA and metabolomics will be reported separately.

### 4.2. VOCs

Total VOC concentrations in urine samples were significantly higher post shift than prior to the shift in Japanese controls and the T-4 group, possibly due to personal activities of the Japanese participants and differences associated with the T-4 aircraft and its operations. Concentrations of VOCs in the urine were measured in ng/100 μg creatinine. The total VOCs in the personal air samples of each group had very low ppb concentrations compared to the acceptable limits recommended by the Japan Society for Occupational Health and Occupational Safety and Health Administration (OSHA), which are not considered to cause adverse health effects in the flight line personnel. Blood levels were also very low, measured in nanogram to microgram concentrations.

Tobacco smoke contains more than 8000 chemicals, including VOCs [24]. The air–blood and air–urinary metabolite relationships of VOCs are known to be affected by smoking habits, age, and gender [25,26]. In this study, the smoking habits of participants had no effect on VOCs in the blood or urine prior to the shift and post shift, and there was no interaction effect between exposure to VOCs and smoking habits.

Only the total VOCs of all samples (air sample, blood, and urine prior to and post shift) for the F-4 group were higher than those of the control group for the same AB. In regard to engine performance, the F-4 engine is a turbojet, while the other aircraft uses a turbofan. The turbofan is more prone to dilution of the combustion gases than the turbojet, as the air in the bypass stream and the combustion gases in the core stream mix at the nozzle. As a result, turbofan engines may have lower total concentrations of VOCs in their exhaust and personal air samples than turbojets, which may also be reflected in the blood and urine. JP-4 jet fuel also contains more short-chain hydrocarbons and aromatics than JetA1 and JP-8. Short-chain hydrocarbons and aromatics (such as benzene) are more volatile than long-chain hydrocarbons. Therefore, short-chain hydrocarbons and aromatics occur more in personal air samples and are more easily absorbed from the lungs, which appears to have been reflected in the blood and urine. Since Jet A is a commercial aviation fuel and JP-8 is the military version of a commercial aviation fuel, the results in this study have potential applications in private and commercial scenarios.

This study measured VOCs in both the blood and urine to confirm exposure, as well as showing uptake and elimination, which provides data for potential physiological modeling. If future studies can sample only one, we advise that urine is much easier to collect and does not require medical personnel (who are needed to draw blood); also, many potential participants are not fond of having their blood drawn.

### 4.3. Noise

The average noise level on flight lines was greater than the OSHA action level (85 dBA) requiring hearing protection and was also greater than the 8 h OSHA (90 dBA) and ACGIH (85 dBA) exposure limits. The time-weighted averages for the sampling time were higher than the OSHA exposure limit when the sampling time was 7 h or greater. The lower time-weighted averages at two Japanese air bases were due to the much shorter average sampling times at those bases.

All exposed personnel wore hearing protection, with many participants wearing both ear plugs and earmuffs. According to the OSHA and NIOSH websites, when worn together, earplugs and earmuffs provide a combined noise reduction rating, which is more protective than either device used alone. With noise level averages in the high 90 dBs and up to 102.2 dB, as shown in Table 5, wearing both ear plugs and earmuffs is recommended to provide a noise reduction to 85 dB. However, wearing both may only provide such protection to 85 dB or less with higher-quality ear plugs plus earmuffs and noise levels less than 100 dB. Hearing protection was sometimes removed for short periods of time (seconds to minutes) when speaking to others. Based on the noise levels around running jet engines, removal after engine start up is not advised. A previous jet fuel study that did not involve noise measurements used controls from a dental clinic. When more controls were needed at one base, controls were again recruited from the dental clinic. Unfortunately, for this study with noise added, that was not a good idea as there is a higher level of noise associated with the equipment used in dentistry.

### 4.4. Audiology

Overall, the audiological histories for JASDF personnel showed less issues with controls than exposed participants. Tinnitus was an issue with F-4 flight line personnel who were working around very loud jet engines, and only one participant reported wearing both ear plugs and earmuffs. Tinnitus was an issue at both USAF bases, although the F-15s were 3% louder than the F-4s (Table 5). There were nine controls at the USAF ABs (four at Kadena and five at Misawa) who reported tinnitus as well. However, only one of the six dental technicians at Kadena AB reported tinnitus, which they experienced occasionally in one ear; they did not wear hearing protection the day we sampled at that base.

The ABR test measures the auditory pathway to the brain, and in this regard, the results of Dreisbach et al. [3] are in line with our T-4, F-16, and F-15 ABR results, where significant differences were seen between exposed and control participants in this Japanese study. Dreisbach et al. [3] studied 24 military fuel specialists working with JP-5 on the flight line. They did not measure noise directly but used a questionnaire. Audiograms and a questionnaire were used to determine potential hearing changes. Although there were no significant differences in audiograms compared to controls, the Amsterdam Inventory for Auditory Disability questionnaire revealed a statistically significant difference in a subgroup of more exposed personnel, who had lower scores in speech intelligibility. Their results suggest that combined exposure to low levels of jet fuel plus aircraft engine noise can affect the central auditory nervous system [3].

Fuente et al. [1] tested fifty-seven Royal Australian Air Force personnel who were selected based on their levels of exposure to jet fuels. Their tests included pure-tone audiometry (1–12 kHz), DPOAE, ABR, and a series of exams for hearing understanding and speech ability. Jet fuel exposure was significantly associated with hearing thresholds at 4 and 8 kHz. Meanwhile, the participants in Japan did not have elevated average hearing thresholds. Fuente et al. found that DPOAEs were significant at 2.8, 4, and 6 kHz, while in Japan, we found significance at 2.9 and 5.7 kHz for T-4, and at 2.2–3.5 kHz plus 4.3 and 5.7 kHz for F-2 in one or both ears. Fuente et al. also found that ABR Wave V latency was significant in the right ear, while in Japan, it was significant in the right ear for F-15 at the fast rate, and in the right ear for F-16 at the Wave V amplitude and Wave III latency at the fast rate, plus for Wave I–V latencies at the slow rate. Overall, there were similar effects seen by Fuente et al. [1] to those in the present study in Japan.

Blair et al. [27] investigated noise and ototoxicant (metals and solvents) exposure and collected pure-tone audiometry results for 2372 US Air Force personnel at a depot-level aircraft maintenance facility. Their threshold shift (STS) criteria results showing hearing changes were significantly worse than for noise exposure alone, which suggested that combined exposure to ototoxicants and noise presents a greater hearing loss risk than just noise. Schaal et al. [28] produced similar significant results based on audiograms of shipyard personnel exposed to noise and both metals and solvents.

Of the 16 ABR endpoints that were significantly different between exposed and control participants, only 2 endpoints had a moderate association according to regression analysis, 1 for noise and 1 for VOCs. Of the 15 out of 28 DPOAE endpoints with associations that were moderate or strong, 10 were for noise and 5 were for VOCs; 4 times, they were in the same ear. Immittance endpoints were only weak, very weak, or showed no associations, so this was the least important of the audiometric tests conducted in this study. Many individual instances of exposure to noise or VOCs showed only minimal associations for significant ABR endpoints, suggesting that a synergistic effect of the combined exposure impacts hearing.

Individual noise exposure data were collected from participants. Because studies have shown the potential for jet fuel to act synergistically with noise, VOCs were measured in the air, blood, and urine to determine the exposure levels in these individuals. In that way, this study showed that personnel working on aircraft are exposed to fuel and noise on the flight line. Since excess noise can impact hearing and VOCs may potentiate hearing issues, audiometric tests were conducted and audiological histories collected to determine if there were any potential hearing effects in flight line personnel. The audiometric test battery used in this study is not the standard routine procedure for monitoring hearing effects. In the tests, hearing appeared to be affected by the combined exposure to noise and jet fuel. Additional studies with larger participant numbers are needed to better understand the possible effects of combined exposure to noise and jet fuel on hearing loss.

## 5. Conclusions

This study explored the characteristics of VOC exposure in the flight line environment for different aircraft types and fuel types. F-4 and JP-4 jet fuels are no longer operational in the JASDF and USAF, and retiring both has made the current flight line environment safer with respect to fuel exposure for maintenance personnel.

Audiometric tests showed significant differences and trends between flight line personnel and control participants for T-4, F-2, F-4, F-15, and F-16 aircraft. The differences were not consistent between aircraft, even though noise levels were comparable for all exposed participants. The consistent finding was a possible trend toward hearing issues for flight line personnel working on military jets in Japan due to the combined exposure to jet fuel and noise on the flight line. Despite levels of VOCs below the exposure limits and the use of hearing protection, a battery of hearing tests (audiograms, DPOAE, immittance, and ABR) showed significant differences and trends towards potential hearing loss.

The combined exposure to noise and jet fuel appears to have a potential health effect related to hearing. Since jet fuel exposure appears to be well below exposure limits, an emphasis on hearing protection is recommended to help prevent hearing issues.

## Figures and Tables

**Table 1:** Assigned noise dosimeter values for the edge noise dosimeter (dB = decibels).

Criterion Level	85 dB
Exchange Rate	3 dB
Upper Limit (used to calculate % dose)	140 dB
Internal Threshold Enable	True
Integrating Threshold	80 dB
Peak Weighting	Z
Projection Time	480 min
Response	Slow
Weighed Frequency Filter	Meter 1 = A Meter 2 = C

**Table 2:** Summary of demographic data for age, experience (years in service and career field), percent tobacco use, and percent female participants.

	Control			Jet Fuel Exposure					Statistically Significant Difference
	CJ	CK	CM	T-4	F-2	F-4	F-15	F-16	
Number	34	22	15	29	7	20	23	18	
Fuel	None			JetA1		JP-4	JP-8		
Age ± SEM *(years) * average ± SEM (standard error of the mean)	27.9 ± 1.0	29.6 ± 1.0	31.3 ± 1.3	26.9 ± 0.8	26.6 ± 1.7	25.2 ± 1.1	22.6 ± 0.6	23.9 ± 0.8	CJ > F-15 CK > F-15 CM > F-15 T-4 > F-15 CK > F-16 CM > F-16
Work experience ± SEM (years in service)	6.0 ± 1.2	8.3 ± 1.1	8.7 ± 1.2	7.2 ± 0.9	7.0 ± 1.8	6.5 ± 1.0	2.5 ± 0.4	3.9 ± 0.6	CK > F-15 CM > F-15 T-4 > F-15 F-2 > F-15 F-4 > F-15
Work experience ± SEM (years in career field)	4.9 ± 1.0	8.0 ± 0.9	5.8 ± 1.2	6.7 ± 0.9	6.4 ± 2.0	6.1 ± 1.1	2.4 ± 0.4	3.5 ± 0.4	not determined
Smokers (%)	9 (26.5)	4 (18.2)	4 (26.7)	13 (44.8)	5 (71.4)	7 (35.0)	9 (39.1)	5 (27.7)	not determined
Female (%)	7 (20.6)	11 (50.0)	6 (40.0)	7 (24.1)	0	0	1(4.3)	3 (16.7)	not determined

**Table 3:** Frequencies of detection and means of volatile organic chemical (VOC) concentrations in personal air samples.

	Control			Jet Fuel Exposure				
	CJ	CK	CM	T-4	F-2	F-4	F-15	F-16
Number	34	22	15	29	7	20	23	17
Fuel	None			JetA1		JP-4	JP-8	
Straight chain alkanes								
n-Hexane (%)	35.3	0	33.3	6.9	57.1	**100**	13.0	35.3
Means ± SEM	0.14 ± 0.04	0	0.88 ± 0.56	0.14 ± 0.10	0.37 ± 0.14	**36.55 ± 20.36**	0.89 ± 0.51	0.43 ± 0.18
n-Heptane (%)	5.9	4.5	6.7	20.7	28.6	**100**	56.5	35.3
Means ± SEM	0.11 ± 0.10	0.01 ± 0.01	0.09 ± 0.09	3.74 ± 1.60	0.15 ± 0.10	**21.41 ± 8.43**	3.04 ± 1.89	0.47 ± 0.24
n-Octane (%)	5.9	0	0	27.6	28.6	**90.0**	**87.0**	35.3
Means ± SEM	0.09 ± 0.08	0	0	0.43 ± 0.15	0.14 ± 0.09	**9.07 ± 3.61**	**8.71 ± 5.76**	0.55 ± 0.30
n-Nonane (%)	14.7	27.3	0	**72.4**	**71.4**	**85.0**	**91.3**	58.8
Means ± SEM	0.08 ± 0.03	0.07 ± 0.02	0	**1.58 ± 0.31**	**0.40 ± 0.11**	**4.84 ± 2.19**	**42.81 ± 26.19**	1.70 ± 0.74
n-Decane (%)	20.6	13.6	0	**89.7**	**85.7**	**65.0**	**91.3**	**82.4**
Means ± SEM	0.29 ± 0.12	0.15 ± 0.10	0	**1.98 ± 0.31**	**0.34 ± 0.06**	**3.26 ± 1.82**	**58.59 ± 32.09**	**1.33 ± 0.49**
n-Undecane (%)	8.8	13.6	6.7	**82.8**	0	55.0	**91.3**	70.6
Means ± SEM	0.03 ± 0.02	1.40 ± 1.01	0.01 ± 0.01	**1.57 ± 0.23**	0	2.70 ± 1.78	**46.87 ± 25.26**	0.82 ± 0.28
n-Dodecane (%)	0	22.7	13.3	**62.1**	0	55.0	**91.3**	82.4
Means ± SEM	0	4.31 ± 3.05	0.02 ± 0.02	**0.94 ± 0.17**	0	2.32 ± 1.72	**26.94 ± 14.82**	0.61 ± 0.19
n-Tridecane (%)	0	36.4	13.3	34.5	0	25.0	**95.7**	76.5
Means ± SEM	0	5.00 ± 3.41	0.03 ± 0.02	0.38 ± 0.11	0	1.55 ± 1.42	**13.03 ± 7.38**	0.48 ± 0.12
n-Tetradecane (%)	14.7	36.4	26.7	0	0	10.0	**91.3**	82.4
Means ± SEM	0.03 ± 0.01	2.66 ± 1.83	0.06 ± 0.03	0	0	0.97 ± 0.93	**4.62 ± 2.59**	0.40 ± 0.12
n-Pentadecane (%)	0	13.6	6.7	3.4	0	10.0	47.8	41.2
Means ± SEM	0	1.09 ± 0.82	0.01 ± 0.01	0.05 ± 0.05	0	0.40 ± 0.39	1.11 ± 0.60	0.18 ± 0.11
Aromatic hydrocarbons								
Benzene (%)	0	0	40.0	3.4	0	**95.0**	26.1	29.4
Means ± SEM	0	0	0.23 ± 0.11	0.04 ± 0.04	0	**7.67 ± 4.28**	0.25 ± 0.14	0.13 ± 0.05
Toluene (%)	**67.6**	50.0	**100**	**75.9**	85.7	**100**	**91.3**	**88.2**
Means ± SEM	**1.19 ± 0.20**	0.36 ± 0.15	**2.58 ± 0.90**	**2.14 ± 0.46**	0.93 ± 0.19	**13.28 ± 4.54**	**2.05 ± 1.06**	**1.27 ± 0.32**
Ethylbenzene (%)	47.1	0	40.0	3.4	0	**60.0**	56.5	41.2
Means ± SEM	0.46 ± 0.11	0	0.25 ± 0.09	0.07 ± 0.07	0	**2.10 ± 0.83**	2.42 ± 1.65	0.29 ± 0.13
m,p-Xylene (%)	47.1	0	80.0	51.7	71.4	**90.0**	**91.3**	**94.1**
Means ± SEM	0.45 ± 0.11	0	0.05 ± 0.12	1.04 ± 0.23	0.49 ± 0.13	**8.34 ± 3.21**	**16.75 ± 10.60**	**1.58 ± 0.48**
o-Xylene (%)	35.3	9.1	13.3	41.4	0	**80.0**	**87.0**	58.8
Means ± SEM	0.18 ± 0.05	0.02 ± 0.01	0.06 ± 0.05	0.27 ± 0.06	0	**1.60 ± 0.64**	**1.48 ± 0.71**	0.70 ± 0.36

Abbreviations: SEM, standard error of the mean. Bold font and squares highlighted in yellow indicate a frequency of detection of VOCs > 60% and mean concentration of VOCs > 1.0 ppb.

**Table 4:** Frequencies of detection and means of VOC concentrations in blood.

	Control			Jet Fuel Exposure				
	CJ	CK	CM	T-4	F-2	F-4	F-15	F-16
Number	34	22	15	29	7	20	22	18
Fuel	None			JetA1		JP-4	JP-8	
Straight chain alkanes								
n-Hexane (%)	8.8	68.2	6.7	6.9	14.3	**70.0**	59.1	11.1
Means ± SEM	0.02 ± 0.01	0.07 ± 0.02	0	0.05 ± 0.05	0.01 ± 0.01	**0.31 ± 0.08**	0.10 ± 0.03	0.15 ± 0.15
n-Heptane (%)	20.6	**90.9**	0	27.6	0	**85.0**	**100**	0
Means ± SEM	0.07 ± 0.03	**0.24 ± 0.07**	0	0.03 ± 0.01	0	**0.36 ± 0.16**	**0.39 ± 0.13**	0
n-Octane (%)	41.2	**77.3**	13.3	10.3	14.3	**95.0**	**100**	11.1
Means ± SEM	0.12 ± 0.04	**0.23 ± 0.07**	0	0.01 ± 0.01	0.01 ± 0.01	**0.45 ± 0.18**	**0.39 ± 0.13**	0.01 ± 0.01
n-Nonane (%)	5.9	40.9	0	**93.1**	100	**95.0**	95.5	11.1
Means ± SEM	0.01 ± 0.01	0.02 ± 0.01	0	**0.11 ± 0.02**	0.07 ± 0.01	**0.11 ± 0.02**	0.07 ± 0.01	0.01 ± 0
n-Decane (%)	0	0	0	0	0	5.0	0	0
Means ± SEM	0	0	0	0	0	0.01 ± 0.01	0	0
n-Undecane (%)	47.1	**100**	66.7	69.0	57.1	**85.0**	**100**	5.6
Means ± SEM	0.08 ± 0.02	**0.11 ± 0.01**	0.07 ± 0.02	0.05 ± 0.01	0.07 ± 0.04	**0.10 ± 0.02**	**0.11 ± 0.01**	0.00 ± 0.00
n-Dodecane (%)	50.0	**100**	**93.3**	24.1	14.3	**100**	**100**	**100**
Means ± SEM	0.11 ± 0.02	**0.20 ± 0.01**	**0.11 ± 0.01**	0.02 ± 0.01	0.03 ± 0.03	**0.17 ± 0.02**	**0.19 ± 0.01**	**0.10 ± 0.01**
n-Tridecane (%)	50.0	**100**	**100**	20.7	14.3	**100**	**100**	100
Means ± SEM	0.16 ± 0.03	**0.40 ± 0.04**	**0.08 ± 0.01**	0.02 ± 0.01	0.01 ± 0.01	**0.34 ± 0.04**	**0.36 ± 0.05**	0.07 ± 0.01
n-Tetradecane (%)	50.0	**95.5**	**100**	6.9	14.3	**100**	**100**	**100**
Means ± SEM	0.23 ± 0.05	**0.29 ± 0.03**	**0.19 ± 0.01**	0.01 ± 0.01	0.02 ± 0.02	**0.58 ± 0.08**	**0.24 ± 0.03**	**0.20 ± 0.01**
n-Pentadecane (%)	35.3	**86.4**	**100**	10.3	0	**100**	54.5	100
Means ± SEM	0.03 ± 0.01	**0.05 ± 0.01**	**0.08 ± 0.01**	0.01 ± 0.01	0	**0.13 ± 0.02**	0.04 ± 0.01	0.09 ± 0.01
Aromatic hydrocarbons								
Benzene (%)	26.5	40.9	46.7	10.3	28.6	**60.0**	59.1	11.1
Means ± SEM	0.02 ± 0.01	0.03 ± 0.01	0.04 ± 0.02	0.01 ± 0.01	0.04 ± 0.03	**0.32 ± 0.11**	0.07 ± 0.02	0.01 ± 0.01
Toluene (%)	**100**	**100**	**100**	**96.6**	**100**	**100**	**100**	**100**
Means ± SEM	**0.36 ± 0.04**	**0.47 ± 0.01**	**0.42 ± 0.04**	**0.15 ± 0.03**	**0.48 ± 0.26**	**0.81 ± 0.06**	**0.48 ± 0.01**	**0.51 ± 0.04**
Ethylbenzene (%)	55.9	**100**	20.0	3.4	14.3	**100**	**100**	83.3
Means ± SEM	0.08 ± 0.02	**0.14 ± 0.01**	0.01 ± 0.01	0.04 ± 0.04	0.05 ± 0.05	**0.54 ± 0.18**	**0.15 ± 0.01**	0.08 ± 0.01
m,p-Xylene (%)	2.9	4.5	0	3.4	14.3	10.0	4.5	0
Means ± SEM	0.01 ± 0.01	0.00 ± 0.00	0	0.11 ± 0.11	0.05 ± 0.05	0.02 ± 0.02	0.00 ± 0.00	0
o-Xylene (%)	41.2	18.2	0	3.4	14.3	90.0	31.8	38.9
Means ± SEM	0.03 ± 0.01	0.01 ± 0	0	0.05 ± 0.05	0.02 ± 0.02	0.07 ± 0.01	0.01 ± 0	0.01 ± 0

Abbreviations: SEM, standard error of the mean. Bold font and squares highlighted in yellow indicate a frequency of detection of VOCs > 60% and concentration of VOCs > 0.1 ng/mL.

**Table 5:** Results for the sound level measurements at the JASDF and USAF Air Bases. The USAF action level is 85 dB for the OSHA exposure limit of 90 dB. See Table 1 for noise dosimeter values.

Participants		Average (dB)	Average (dB)	Actual TWA for Time of Sampling (dB)	Actual TWA for Time of Sampling (dB)	Sampling Time (h)	Plane/Jet Fuel
		Meter 1	Meter 2	Meter 1	Meter 2		
Hyakuri Flight Line	Average	98.9	100.2	98.8	100.1	8.2	F-4
	SEM	0.5	0.5	0.7	0.7	0.6	JP-4
Matsushima Flight Line	Average	98.0	91.0	94.4	85.1	3.6	F-2
	SEM	0.7	0.8	0.5	0.5	0.2	Jet A
Hamamatsu Flight Line	Average	97.5	92.5	92.4	84.1	2.5	T-4
	SEM	0.7	0.4	0.7	0.4	0.04	Jet A
JASDF Controls	Average	58.2	67.9	55.7	65.4	4.7	N/A
	SEM	1.8	1.6	1.8	1.6	0.2	
JASDF Controls—Iruma AB	Average	48.3	25.5	48.1	25.1	7.6	N/A
	SEM	1.4	6.9	1.4	6.9	0.1	
Kadena Flight Line	Average	102.2	101.9	103.0	102.6	9.7	F-15
	SEM	0.9	0.9	1.0	1.0	0.3	JP-8
Kadena Controls	Average	66.3	75.2	65.7	74.7	7.0	N/A
	SEM	1.8	2.1	1.8	2.1	0.1	
Misawa Flight Line	Average	90.7	93.6	90.6	93.5	7.8	F-16
	SEM	2.2	1.7	2.2	1.7	0.1	JP-8
Misawa Controls	Average	69.2	74.2	69.3	78.7	8.2	N/A
	SEM	1.6	5.1	1.6	1.5	0.2	

TWA = time-weighted average; SEM = standard error of the mean; N/A = not applicable.

**Table 6:** ABR slow-rate and fast-rate results for T-4 participants with values in milliseconds. W = wave; Lat = latency; * amplitude/latency ratios (µV/ms; termed response magnitudes); ^+^ Controls = Iruma, Hyakuri, and Yokota; values are means ± standard error of the mean.

Ear & Rate	W I Lat	W V Lat	I-III Lat	III-V Lat	I-V Lat	W I Amp	W V Amp	W V/W 1 Amps	Amp/latency * Wave 1	Amp/latency Wave V
Right slow										
Control ^+^ n = 34	1.67 0.036	5.68 0.041	2.21 0.046	1.84 0.021	4.03 0.035	0.24 0.023	0.45 0.027	2.55 0.28	0.15 0.016	0.080 0.005
T-4 n = 28	1.60 0.018	5.73 0.034	2.29 0.051	1.89 0.017	4.14 0.031	0.21 0.017	0.38 0.030	2.40 0.39	0.13 0.012	0.07 0.005
*p*	0.11	0.37	0.25	0.074	0.022	0.36	0.085	0.75	0.34	0.19
Left slow										
Control ^+^ n = 34	1.58 0.018	5.70 0.035	2.22 0.031	1.87 0.026	4.09 0.039	0.26 0.019	0.43 0.022	2.13 0.26	0.17 0.013	0.08 0.004
T-4 n = 29	1.59 0.018	5.73 0.030	2.29 0.035	1.88 0.021	4.14 0.025	0.19 0.016	0.39 0.028	2.26 0.23	0.12 0.011	0.07 0.005
*p*	0.69	0.53	0.14	0.77	0.30	0.0106	0.207	0.70	0.004	0.10
Right fast										
Control ^+^ n = 34		6.21 0.04					0.27 0.01			0.043 0.002
T-4 n = 29		6.25 0.05					0.24 0.02			0.038 0.003
*p*		0.55					0.17			0.188
Left fast										
Control ^+^ n = 34		6.14 0.03					0.27 0.01			0.044 0.002
T-4 n = 29		6.25 0.05					0.25 0.02			0.040 0.003
*p*		0.067					0.39			0.35

**Table 7:** DPOAE results (in dB) for F-2 flight line participants across test frequencies (in bolded Hz). ^+^ Controls = Iruma, Hyakuri, and Yokota; dB values are means ± standard error of the mean.

Ear								
Right	**2000.0**	**2198.0**	**2416.0**	**2656.0**	**2920.0**	**3210.0**	**3529.0**	**3880.0**
Control ^+^ n = 32	20.7 1.77	21.2 1.21	20.5 0.98	22.6 1.03	22.8 1.02	23.6 1.05	25.3 1.11	25.9 1.16
F-2 n = 7	16.69 2.08	14.96 1.78	15.20 1.60	14.23 1.56	16.24 2.05	16.73 2.11	19.37 1.96	23.21 2.05
*p*	0.31	0.029	0.022	0.0009	0.0085	0.0078	0.026	0.32
Left								
Control ^+^ n = 32	21.61 1.03	20.70 0.93	20.79 1.00	22.35 1.08	23.31 1.05	23.01 1.21	25.06 1.03	26.72 1.04
F-2 n = 7	19.23 1.94	17.41 1.60	16.44 1.53	15.44 1.93	15.40 1.76	15.80 1.17	18.93 2.07	22.90 2.16
*p*	0.33	0.13	0.062	0.008	0.002	0.01	0.015	0.13
Right	**4265.0**	**4688.0**	**5154.0**	**5666.0**	**6229.0**	**6847.0**	**7527.0**	**8275.0**
Control^+^ n = 32	27.6 1.01	27.4 1.06	29.6 1.23	30.3 1.35	28.3 1.58	29.0 1.48	22.5 1.29	22.7 1.25
F-2 n = 7	21.79 1.70	21.66 2.19	26.09 1.38	28.63 1.19	26.79 1.55	26.23 2.00	23.24 2.06	21.60 1.59
*p*	0.016	0.027	0.21	0.57	0.67	0.41	0.80	0.70
Left								
Control^+^ n = 32	27.41 1.03	27.94 1.11	28.98 1.25	30.31 1.21	29.49 1.22	28.58 1.37	22.86 1.28	21.01 1.26
F-2 n = 7	22.10 2.28	22.79 2.17	23.71 1.98	24.61 1.59	24.43 2.04	25.66 1.56	22.43 2.44	21.36 2.36
*p*	0.036	0.054	0.07	0.043	0.08	0.34	0.89	0.91

**Table 8:** ABR results for Hyakuri AB F-4 flight line participants with values in milliseconds. W = wave; Lat = latency; * amplitude/latency ratios (µV/ms; termed response magnitudes); ^+^ Controls = Iruma, Hyakuri, and Yokota; values are means ± standard error of the mean.

Ear & Rate	W I Lat	W V Lat	I-III Lat	III-V Lat	I-V Lat	W I Amp	W V Amp	W V/W 1 Amps	Amp/latency * Wave 1	Amp/latency Wave V
Right slow										
Control ^+^ n = 34	1.67 0.036	5.68 0.041	2.21 0.046	1.84 0.021	4.03 0.035	0.24 0.023	0.45 0.027	2.55 0.28	0.15 0.016	0.080 0.005
F-4 n = 20	1.57 0.031	5.59 0.042	2.25 0.065	1.82 0.020	3.97 0.048	1.09 0.59	0.51 0.023	2.56 0.48	0.63 0.33	0.091 0.005
*p*	0.061	0.14	0.61	0.52	0.30	0.067	0.14	0.98	0.062	0.15
Left slow										
Control ^+^ n = 34	1.58 0.018	5.70 0.035	2.22 0.031	1.87 0.026	4.09 0.039	0.26 0.019	0.43 0.022	2.13 0.26	0.17 0.013	0.08 0.004
F-4 n = 20	1.57 0.039	5.62 0.046	2.21 0.031	1.85 0.029	4.06 0.036	0.21 0.023	0.44 0.019	2.49 0.34	0.14 0.019	0.078 0.004
*p*	0.79	0.18	0.83	0.62	0.61	0.10	0.76	0.40	0.17	0.87

**Table 9:** Audiogram data for T-4 participants measured in decibels Hearing level (dB HL) at frequencies in HZ between 500 and 6000.

Ear	500 HZ	1000 HZ	2000 HZ	3000 HZ	4000 HZ	6000 HZ
Right	20.00	9.83	6.55	5.69	6.03	16.38
T-4	1.36	1.12	1.00	1.16	1.50	1.66
n = 29						
Left	15.52	9.14	7.59	6.55	5.52	16.72
T-4	1.45	1.11	0.95	1.17	1.43	2.29
n = 29						

Values are means ± standard error of the mean, in bolded Hz.

**Table 10:** Audiogram data for F-2 participants measured in decibels Hearing level (dB HL) at frequencies in HZ between 500 and 6000.

Ear	500 HZ	1000 HZ	2000 HZ	3000 HZ	4000 HZ	6000 HZ
Right	17.86	7.14	7.14	1.43	7.86	16.43
F-2	1.84	1.84	2.14	1.80	3.91	2.61
n = 7						
Left	10.71	6.43	8.57	2.86	7.14	18.57
F-2	1.70	1.80	2.10	2.14	3.43	4.97
n = 7						

Values are means ± standard error of the mean, in bolded Hz.

**Table 11:** Immittance data for Misawa AB F-16 flight line participants.

Ear	SC	Peak P	ECV	AR 500	AR 1000	AR 2000	AR 500 Contra	AR 1000 Contra	AR 2000 Contra
Control	0.82	−4.13	1.35	85.00	87.14	88.00	95.77	92.73	93.21
Right	0.15	2.04	0.08	1.38	1.55	1.60	0.96	0.79	1.13
n	15	15	15	12	14	15	13	11	14
F-16	0.78	4.61	1.44	90.29	90.59	88.82	95.00	97.69	96.00
Right	0.06	2.52	0.07	1.09	0.73	1.18	0.83	0.72	1.21
n	18	18	18	17	17	17	9	13	15
*p*	0.8	0.014	0.38	0.005	0.04	0.68	0.57	0.0001	0.11
Control	0.72	−4.80	1.22	85.38	86.54	88.21	92.08	93.57	91.15
Left	0.12	2.10	0.07	1.44	1.73	1.86	1.56	1.22	1.29
n	15	15	15	13	13	14	12	14	13
F-16	0.71	−0.28	1.32	89.71	88.82	88.13	95.00	97.31	95.36
Left	0.07	1.86	0.06	1.39	1.10	0.98	1.51	1.22	0.98
n	18	18	18	17	17	17	11	13	14
*p*	0.94	0.12	0.3	0.04	0.26	0.97	0.19	0.04	0.014

SC = static compliance (cc); Peak P = peak pressure (daPa); ECV = equivalent ear canal volume (cc); AR = acoustic reflex (dB); Contra = contralateral; values are means ± standard error of the mean.

**Table 12:** ABR slow-rate and fast-rate results for Misawa AB F-16 flight line participants with values in milliseconds. W = wave; Lat = latency; * amplitude/latency ratios (µV/ms; termed response magnitudes); values are means ± standard error of the mean.

Ear & Rate	W I Lat	W IIILat	W V Lat	I-III Lat	III-V Lat	I-V Lat	W I Amp	W V Amp	W V/W 1 Amps	* Amp/latency Wave 1	Amp/latency Wave V
Right slow											
Control n = 15	1.61 0.03	3.86 0.048	5.69 0.05	2.25 0.04	1.83 0.02	4.08 0.03	0.29 0.03	0.47 0.04	1.76 0.18	0.18 0.02	0.082 0.008
F-16 n = 18	1.62 0.02	3.72 0.027	5.65 0.03	2.10 0.026	1.95 0.03	4.04 0.03	0.33 0.04	0.40 0.03	1.50 0.25	0.21 0.02	0.070 0.005
*p*	0.77	0.013	0.45	0.003	0.004	0.37	0.38	0.18	0.43	0.33	0.22
Left slow											
Control n = 15	1.59 0.02	3.86 0.05	5.70 0.05	2.27 0.04	1.84 0.04	4.11 0.04	0.28 0.03	0.48 0.06	2.18 0.46	0.18 0.02	0.086 0.011
F-16 n = 18	1.62 0.02	3.80 0.03	5.66 0.03	2.17 0.034	1.87 0.04	4.03 0.03	0.31 0.04	0.36 0.03	1.56 0.29	0.19 0.02	0.063 0.005
*p*	0.37	0.28	0.48	0.07	0.59	0.14	0.52	0.05	0.25	0.74	0.059
Right fast											
Control n = 15			6.14 0.06					0.28 0.02			0.05 0.00
F-16 n = 18			6.11 0.05					0.22 0.02			0.04 0.003
*p*			0.70					0.04			0.057
Left fast											
Control n = 15			6.12 0.07					0.30 0.04			0.05 0.01
F-16 n = 18			6.13 0.05					0.23 0.02			0.04 0.004
*p*			0.90					0.08			0.08

**Table 13:** ABR slow- and fast-rate results for Kadena AB F-15 flight line participants with values in milliseconds. W = wave; Lat = latency; * amplitude/latency ratios (µV/ms; termed response magnitudes); values are means ± standard error of the mean.

Ear & Rate	W I Lat	W IIILat	W V Lat	I-III Lat	III-V Lat	I-V Lat	W I Amp	W V Amp	W V/W 1 Amps	* Amp/latency Wave 1	Amp/latency Wave V
Right slow											
Control n = 22	1.61 0.03	3.86 0.048	5.69 0.05	2.25 0.04	1.83 0.02	4.08 0.03	0.29 0.03	0.47 0.04	1.76 0.18	0.18 0.02	0.082 0.008
F-15 n = 23	1.62 0.02	3.72 0.027	5.65 0.03	2.10 0.026	1.95 0.03	4.04 0.03	0.33 0.04	0.40 0.03	1.50 0.25	0.21 0.02	0.070 0.005
*p*	0.77	0.013	0.45	0.003	0.004	0.37	0.38	0.18	0.43	0.33	0.22
Left slow											
Control n = 22	1.59 0.02	3.86 0.05	5.70 0.05	2.27 0.04	1.84 0.04	4.11 0.04	0.28 0.03	0.48 0.06	2.18 0.46	0.18 0.02	0.086 0.011
F-15 n = 23	1.62 0.02	3.80 0.03	5.66 0.03	2.17 0.034	1.87 0.04	4.03 0.03	0.31 0.04	0.36 0.03	1.56 0.29	0.19 0.02	0.063 0.005
*p*	0.37	0.28	0.48	0.07	0.59	0.14	0.52	0.05	0.25	0.74	0.059
Right fast											
Control n = 22			5.95 0.04					0.26 0.02			0.04 0.00
F-15 n = 23			6.07 0.05					0.31 0.03			0.05 0.01
*p*			0.02					0.20			0.12
Left fast											
Control n = 22			6.05 0.06					0.26 0.02			0.05 0.00
F-15 n = 23			6.05 0.05					0.31 0.02			0.05 0.00
*p*			1.0					0.12			0.67

**Table 14:** Regression analysis of significant audiometric endpoints versus noise and VOCs.

**Significant Audiological Test**			**Noise**		
**ABR**	**Aircraft**	**r**	**r^2^**	***p* Value**	**Comments**
Wave I-III Latency, right ear, slow rate	F-16	0.5	0.25	0.03	moderate association (r 0.4–0.6), not very linear but significance between variables
Wave III-V Latency, right ear, slow rate	F-16	0.04	0.001	0.89	no association
Wave V Amp, right ear, fast rate	F-16	0.23	0.05	0.35	weak association
T-4 I-V Latency right ear slow	T-4	0.13	0.02	0.52	very weak association
Wave V Latency, right ear, fast rate	F-15	0.01	0.0004	0.96	no association
6 additional regressions	2 F-16; 4 T-4				No apparent associations
**DPOAE**					
2656 Hz, right ear	F-2	0.81	0.66	0.03	very strong association (r > 0.8), weak linearity, significance between variables
2656 Hz, left ear	F-2	0.32	0.1	0.48	weak association
2920 Hz, right ear	F-2	0.72	0.52	0.07	strong association, weak linearity, almost significant
2920 Hz, left ear	F-2	0.72	0.51	0.07	strong association, weak linearity, almost significant
3210 Hz, right ear	F-2	0.55	0.31	0.2	moderate association
3210 Hz, left ear	F-2	0.73	0.53	0.06	strong association, weak linearity, almost significant
3529 Hz, right ear	F-2	0.84	0.71	0.02	very strong association, weak linearity, significance between variables
3529 Hz, left ear	F-2	0.73	0.53	0.06	strong association, weak linearity, almost significant
4265 Hz, right ear	F-2	0.75	0.57	0.05004	strong association, weak linearity, essentially significance between variables
4265 Hz, left ear	F-2	0.64	0.41	0.12	strong association
5666 Hz, left ear	F-2	0.54	0.29	0.21	moderate association
2416 Hz and 4688 Hz right ear	F-2				weak associations
2198 Hz, right ear	F-2				very weak association
**Immittance**					
Acoustic Reflex 500, right ear	F-16				very weak association
Acoustic Reflex 500, left ear	F-16				no association
Acoustic Reflex 1000, right ear	F-16				very weak association
AR 1000 Contra, right ear	F-16				weak associations
AR 1000 Contra left ear	F-16				no association
AR 2000 Contra, left ear	F-16				very weak association
**Significant Audiological Test**			**VOCs**		
**ABR**	**Aircraft**	**r**	**r^2^**	***p* Value**	**Comments**
Wave I-III Latency, right ear, slow rate	F-16	0.19	0.04	0.46	very weak association r <0.2)
Wave III-V Latency, right ear, slow rate	F-16	0.3	0.09	0.24	weak association (r 0.2-0.4)
Wave V Amp, right ear, fast rate	F-16	0.14	0.02	0.59	very weak association
T-4 I-V Latency right ear slow	T-4	0.03	0.001	0.86	no association
Wave V Latency, right ear, fast rate	F-15	0.403	0.16	0.06	moderate association, not very linear, almost significant
6 additional regressions	2 F-16; 4 T-4				No apparent associations
**DPOAE**					
2656 Hz, right ear	F-2	0.13	0.02	0.78	very weak association
2656 Hz, left ear	F-2	0.76	0.57	0.048	strong association (r 0.6-0.8), weak linearity, significance between variables
2920 Hz, right ear	F-2	0.32	0.1	0.48	weak association
2920 Hz, left ear	F-2	0.55	0.31	0.2	moderate association, weak linearity, not significant
3210 Hz, right ear	F-2	0.25	0.06	0.59	weak association
3210 Hz, left ear	F-2	0.07	0.004	0.89	no association
3529 Hz, right ear	F-2	0.25	0.06	0.58	weak association
3529 Hz, left ear	F-2	0.38	0.15	0.4	weak association
4265 Hz, right ear	F-2	0.5	0.25	0.26	strong association
4265 Hz, left ear	F-2	0.51	0.26	0.24	moderate association
5666 Hz, left ear	F-2	0.6	0.36	0.16	strong association
2416 Hz and 4688 Hz right ear	F-2				weak associations
2198 Hz, right ear	F-2				very weak association
**Immittance**					
Acoustic Reflex 500, right ear	F-16				no association
Acoustic Reflex 500, left ear	F-16				weak associations
Acoustic Reflex 1000, right ear	F-16				weak associations
AR 1000 Contra, right ear	F-16				very weak association
AR 1000 Contra left ear	F-16				no association
AR 2000 Contra, left ear	F-16				weak associations

## Data Availability

The VOC data can be found in the Supplementary Materials for this manuscript and in Orui et al. [21] as a technical report to the Defense Technology Information Center (DTIC). The USAF data can be found in the Supplementary Materials for this manuscript and in the USAF technical report to the DTIC, as per Mattie et al. [18].

## Supplementary Materials

The following supporting information can be downloaded at https://www.mdpi.com/article/10.3390/toxics13020121/s1, Figure S1: Comparison of the total VOCs in blood, urine prior to a shift, and urine post shift in smokers and non-smokers. Table S1: Number of participants and sampling date at each air base. Table S2: Summary of initial demographic data for age, experience (years in service and time in career field), gender, hobbies involving VOCs, tobacco use, and caffeine intake by JASDF Air Bases. Table S3: Summary of initial demographic data for age, experience (years in service and time in career field), gender, hobbies involving VOCs, tobacco use, and caffeine intake by USAF Air Bases. Table S4: Permissible exposure limits of VOCs. Table S5: The calculated additive mixture formula values according to the Japan Society for Occupational Health (JSOH, 2022) and the Occupational Safety and Health Administration (OSHA, 2022) exposure limits for each JASDF Aeromedical Laboratory group. Table S6: Results of the Audiological History questionnaires for JASDF Air Bases. Table S7: Results of Audiological History questionnaires for USAF Air Bases. Table S8: Immittance data for T-4 participants. Table S9: DPOAE data for T-4 participants with values in Hz. Table S10: Immittance data for F-2 participants. Table S11: ABR results for F-2 participants with values in milliseconds. Table S12: Immittance data for Hyakuri AB F-4 participants. Table S13: DPOAE data for Hyakuri AB F-4 participants with values in Hz. Table S14: Audiogram data for F-16 participants measured in decibels. Hearing level, dB HL. Table S15: DPOAE data for Misawa AB F-16 participants with values in Hz.
