# Supplementary material for: Comparison of Operational Jet Fuel and Noise Exposure for Flight Line Personnel at Japanese and United States Air Bases in Japan
Source: Toxics. 2025 Feb 5;13(2):121. doi: 10.3390/toxics13020121 (PMC11860679; doi:10.3390/toxics13020121)
Supplement: Supplementary file 1 [file toxics-13-00121-s001.zip › toxics-3377801-supplementary.pdf]

Supplemental File

Table S1. Number of participants and sampling date at each air base

Date	Air Base	Aircrafts	Jet fuel	Assignment	Number of Participants
30 May - 8 June 2018	Hyakuri JASDF	F-4	JP-4	The 301st SQ<br>The 302nd SQ	20
		Control		7th Air Wing Air Base Group (Medical workers)	2
11 June - 12 June 2018	Yokota JASDF	Control		Operations Support Wing (Medical workers)	5
13 June - 15 June 2018	Iruma JASDF	Control		Aeromedical Laboratory (Medical workers)	11
19 March - 10 April 2019	Kadena USAF	F-15	JP-8	The44th SQ<br>The 67th SQ	23
		Control		The 18th Med Gr (Medical workers)	22
8 November - 12 November 2021	Matsushima JASDF	T-4	JetA1	The 21st SQ	7
		F-2		The 11th SQ	7
6 December - 10 December 2021	Hamamatsu JASDF	T-4	JetA1	The 31st SQ<br>The 32nd SQ	22
11 July - 22 July 2022	Misawa USAF	F-16	JP-8	The14th SQ	18
		Control		The 35th Med Gr (Medical workers)	15
21 October – 27 October 2022	Iruma JASDF	Control		Aeromedical Laboratory (Medical workers)	16
Total number					168

Table S2. Summary of initial demographic data for age, experience (years in service and time in career field), gender, hobbies involving VOCs, tobacco use and caffeine intake by JASDF Air Bases

Base / Subjects	Rank	Career Field by Name	Years of service	Age	Gender	Time in career field (Yrs)	Hobbies involving VOCs	Tobacco use	Caffeinated drinks (based on 8 oz)
Hyakuri Exposed	Airman = 12<br>SSgt = 7<br>TSgt = 1	Aircraft Maintenance	6.5 ± 1.0	25.2 ± 1.1	Male = 20	6.1 ± 1.1	0.2 ± 0.1	0.4 ± 0.1	1.4 ± 0.3
Hyakuri Controls*	Enlisted = 10<br>Officer = 7<br>civilian = 1	Medical Corps	9.0 ± 2.0	29.2 ± 1.7	Male = 14<br>Female = 4	6.9 ± 1.8	0	0.3 ± 0.1	#1.9 ± 0.4
Matushima Exposed	AIC = 3<br>SSgt = 10<br>2nd LT = 1	Aircraft Maintenance	8.8 ± 1.1	28.1 ± 1.0	Male = 13<br>Female = 1	8.4 ± 1.2	0	0.4 ± 0.1	0.5 ± 0.2
Hamamatsu Exposed	AIC = 9<br>SSgt = 12<br>TSgt = 1	Aircraft Maintenance	6.2 ± 1.1	26.0 ± 1.0	Male = 16<br>Female = 6	5.8 ± 1.0	0.3 ± 0.2	0.5 ± 0.1	1.1 ± 0.2
Iruma Controls+	Enlisted = 8<br>2nd LT = 4<br>civilian = 4	Medical Corps; Researcher; Pharmacist	2.7 ± 0.5	26.4 ± 0.8	Male = 13<br>Female = 3	2.7 ± 0.5	0.1 ± 0.1	0.2 ± 0.1	1.2 ± 0.3

Table S3. Summary of initial demographic data for age, experience (years in service and time in career field), gender, hobbies involving VOCs), tobacco use and caffeine intake by USAF Air Bases

Base / Subjects	Rank	Career Field by Name	Years of service	Age	Gender	Time in career field (Yrs)	Hobbies involving VOCs	Tobacco use	Caffeinated drinks (based on 8 oz)
Kadena Exposed	Airman = 2<br>AIC = 12<br>SRA = 8<br>SSgt = 1	Crew Chief= 18<br>Avionics = 4<br>elect & environ = 1	2.5 ± 0.4	22.6 ± 0.6	Male = 22<br>Female = 1	2.4 ± 0.4	0.1 ± 0.1	0.4 ± 0.1	1.1 ± 0.3
Kadena Control	Enlisted = 21<br>Officer = 1	BE Tech;<br>Pub Health;<br>Dental	8.3 ± 0.9	29.6 ± 4.7	Male = 11<br>Female = 11	8.0 ± 0.9	0.05 ± 0.2	0.05 ± 0.04	1.0 ± 0.2
Misawa Exposed	AIC = 6<br>SRA = 7<br>SSgt = 4<br>TSgt = 1	Crew Chief = 7<br>Armament = 3<br>AC Maint. = 3<br>AGE = 3<br>Specialist = 2	3.9 ± 0.6	23.9 ± 0.8	Male = 15<br>Female = 3	3.5 ± 0.4	0.06 ± 0.06	0.3 ± 0.1	1.7 ± 0.5
Misawa Control	Enlisted = 14<br>Officer = 1	Flight Medicine<br>BE Tech	8.7 ± 1.2	31.3 ± 1.4	Male = 9<br>Female = 6	5.8 ± 1.2	0.07 ± 0.07	0.2 ± 0.1	1.4 ± 0.3

Table S4. Permissible exposure limits of VOCs

VOCs (ppm)	Japan Society for Occupational Health (JSOH: 2022)	Occupational Safety and Health Administration (OSHA: 2022)
n-Hexane	40	500
n-Heptane	200	500
n-Octane	300	-
n-Nonane	200	-
n-Decane	-	-
n-Undecane	-	
n-Dodecane	-	-
n-Tridecane	-	-
n-Tetradecane	-	-
n-Pentadecane	-	-
Benzene	-	1
Toluene	50	200
Ethylbenzene	20	100
Xylene (o-,m-,p-isomers)	50	100

Table S5. The calculated additive mixture formula values by Japan Society for Occupational Health (JSOH, 2022) and Occupational Safety and Health Administration (OSHA, 2022) exposure limits for each JASDF Aeromedical Laboratory group

Groups by Base and Aircraft	Additive Mixture Formula: JSOH	Additive Mixture Formula: OSHA
Non-exposed controls in JASDF	7x10-5	2 x10-5
Non-exposed controls in Kadena	1 x10-5	<1 x10-5
Non-exposed controls in Misawa	1x10-4	2 x10-4
Jet A1 exposed T-4 flight line crews in Matsushima and Hamamatsu	1 x10-4	7 x10-5
Jet A1 exposed F-2 flight line crews in Matsushima	4 x10-5	1 x10-5
JP-4 exposed F-4 flight line crews in Hyakuri	2 x10-3	8 x10-3
JP-8 exposed F-15 flight line crews in Kadena	1 x10-3	5 x10-4
JP-8 exposed F-16 flight line crews in Misawa	1 x10-4	2 x10-4

Table S6. Results of the Audiological History questionnaires for JASDF Air Bases

Base (Mean ± SEM)	n	Hearing loss - temporary	Chronic or bothersome tinnitus	Ear infections /drainage, or surgeries	Spinning dizziness /vertigo, or dizziness with head/neck movement	Ear pain or feeling of fullness	Family history of hearing loss	Chronic or recurrent medical condition	**None of these**	Taking herbal supplement	History of noise exposure off duty or recreationally (x out of n)
Hyakuri Exposed	20	0.1 ± 0.07	0.37 ± 0.11	0	0.15 ± 0.08	0.1 ± 0.07	0.05 ± 0.05	0	0.45 ± 0.11	0.1 ± 0.07	
n of 20		2	7	0	3	2	1	0	9	2	9
Hyakuri Controls	18	0.11 ± 0.08	0	0.11 ± 0.08	0	0.06 ± 0.06	0	0	0.89 ± 0.08	0.28 ± 0.11	
n of 18		2	0	2	0	1	0	0	16	5	1
Matushima Exposed	14	0	0	0	0	0	0.07 ± 0.07	0	0.93 ± 0.07	0.07 ± 0.07	
n of 14		0	0	0	0	0	1	0	13	1	3
Hamamatsu Exposed	22	0.05 ± 0.05	0	0	0.05 ± 0.05	0.05 ± 0.05	0.05 ± 0.05	0	0.86 ± 0.07	0.32 ± 0.10	
n of 22		1	0	0	1	1	1	0	19	7	3
Iruma Controls	16	0	0.06 ± 0.06	0.06 ± 0.06	0.06 ± 0.06	0	0	0	0.81 ± 0.10	0.19 ± 0.10	
n of 16		0	1	1	1	0	0	0	13	3	1

SEM =standard error of the mean

Table S7. Results of Audiological History questionnaires for USAF Air Bases

Base (Mean ± SEM)	n	Hearing loss - temporary	Chronic or bothersome tinnitus	Ear infections /drainage, or surgeries	Spinning dizziness /vertigo, or dizziness with head/neck movement	Ear pain or feeling of fullness	Family history of hearing loss	Chronic or recurrent medical condition	**None of these**	Taking herbal supplement	History of noise exposure off duty or recreationally (x out of n)
Kadena Exposed	23	0.04 ± 0.04	0.26 ± 0.09	0	0.04 ± 0.04	0.09 ± 0.06	0.17 ± 0.08	0	0.57 ± 0.11	0.09 ± 0.06	
n of 23		1	6	0	1	2	4	0	13	2	12
Kadena Control	22	0.09 ± 0.06	0.18 ± 0.08	0.14 ± 0.07	0.09 ± 0.06	0.05 ± 0.05	0	0.09 ± 0.06	0.64 ± 0.10	0.27 ± 0.10	
n of22		2	4	3	2	1	0	2	14	6	7
Misawa Exposed	18	0.11 ± 0.08	0.56 ± 0.12	0.11 ± 0.08	0.11 ± 0.08	0.11 ± 0.08	0	0	0.33 ± 0.11	0.22 ± 0.10	
n of 18		2	10	2	2	2	0	0	6	4	9
Misawa Control	15	0	0.33 ± 0.13	0.13 ± 0.09	0.07 ± 0.07	0.33 ± 0.13	0.13 ± 0.09	0.33 ± 0.13	0.27 ± 0.12	0.33 ± 0.13	
n of 15		0	5	2	1	5	2	5	4	5	8

SEM = standard error of the mean

Table S8. Immittance data for T-4 flight line participants

Subject										
Control		SC	Peak P	ECV	AR500-I	AR1000-I	AR2000-I	AR500-C	AR1000-C	AR2000-C
Right Ear	Mean	0.58	-5.21	1.29	86.1	87.3	88.3	95.2	94.0	94.1
all Iruma + HY +	STD	0.26	12.81	0.33	6.3	5.7	5.9	5.6	4.7	5.4
Yokota	SEM	0.04	2.20	0.06	1.2	1.1	1.1	1.1	0.9	1.0
	n	34	34	34	28	28	30	26	27	29
T-4	Mean	0.75	-2.57	1.24	87.8	88.6	89.0	94.8	94.8	95.9
Right Ear	STD	0.35	10.43	0.28	6.4	4.7	4.9	6.1	5.7	4.7
	SEM	0.07	1.97	0.05	1.2	0.9	0.9	1.3	1.2	1.0
	n	28	28	28	29	28	29	22	22	23
		p=0.03	p=0.38	p=0.53	p=0.32	p=0.36	p=0.62	p=0.81	p=0.59	p=0.21
Conrol	Mean	0.67	-8.00	1.27	85.5	87.0	88.9	92.4	93.8	92.7
Left Ear	STD	0.30	12.12	0.32	5.4	5.2	5.9	6.6	5.9	5.9
all Iruma + HY +	SEM	0.05	2.08	0.06	1.0	0.9	1.0	1.4	1.2	1.2
Yokota	n	34	34	34	32	32	32	21	24	24
T-4	Mean	0.82	-3.54	1.20	88.6	85.5	89.7	94.3	93.6	95.2
left ear	STD	0.49	8.17	0.30	7.1	15.8	5.2	5.1	4.8	4.2
	SEM	0.09	1.54	0.06	1.3	3.0	1.0	1.1	1.0	0.9
	n	28	28	28	28	28	29	21	21	22
		p=0.14	p=0.10	p=0.38	p=0.50	p=0.61	p=0.58	p=0.30	p=0.90	p=0.11

SC=static compliance (cc); Peak P=peak pressure (daPa); ECV=equivalent ear canal volume (cc); AR=acoustic reflex (dB); C=contralateral

Table S9. DPOAE data for T-4 flight line participants with values in Hz

Control	Ear	2000	2198	2416	2656	2920	3210	3529	3880	4265	4688	5154	5666	6229	6847	7527	8275
Mean	Right	20.7	21.2	20.5	22.6	22.8	23.6	25.3	25.9	27.6	27.4	29.6	30.3	28.3	29.0	22.5	22.7
STD		10.0	6.9	5.5	5.8	5.7	5.9	6.3	6.6	5.7	6.0	7.0	7.6	8.9	8.4	7.3	7.1
SEM		1.8	1.2	1.0	1.0	1.0	1.0	1.1	1.2	1.0	1.1	1.2	1.3	1.6	1.5	1.3	1.2
n		32	32	32	32	32	32	32	32	32	32	32	32	32	32	32	32
T-4																	
Mean	Right	21.9	20.7	19.4	21.2	22.1	21.4	22.8	24.7	26.4	27.0	26.4	26.8	26.9	26.0	23.4	22.9
STD		6.8	6.7	5.3	6.6	4.9	5.1	5.4	5.0	4.6	5.4	5.3	5.9	8.0	10.1	6.6	7.4
SEM		1.3	1.2	1.0	1.2	0.9	0.9	1.0	0.9	0.9	1.0	1.0	1.1	1.5	1.9	1.2	1.4
n		29	29	29	29	29	29	29	29	29	29	29	29	29	29	29	29
		p=0.59	p=0.78	p=0.43	p=0.38	p=0.61	p=0.13	p=0.10	p=0.43	p=0.37	p=0.79	p=0.83	p=0.048	p=0.52	p=0.21	p=0.62	p=0.91
Control																	
Mean	Left	21.6	20.7	20.8	22.4	23.3	23.0	25.1	26.7	27.4	27.9	29.0	30.3	29.5	28.6	22.9	21.0
STD		5.8	5.3	5.7	6.1	5.9	6.9	5.8	5.9	5.8	6.3	7.1	6.9	6.9	7.8	7.2	7.1
SEM		1.0	0.9	1.0	1.1	1.0	1.2	1.0	1.0	1.0	1.1	1.2	1.2	1.2	1.4	1.3	1.3
n		32	32	32	32	32	32	32	32	32	32	32	32	32	32	32	32
T-4																	
Mean	Left	20.0	19.9	19.6	19.7	19.9	21.7	23.4	26.0	26.6	28.2	28.0	29.0	27.9	27.9	23.9	23.0
STD		6.4	5.7	5.0	5.1	5.6	5.0	4.4	4.2	4.7	4.2	4.5	4.6	5.0	5.5	4.7	4.8
SEM		1.2	1.1	0.9	0.9	1.0	0.9	0.8	0.8	0.9	0.8	0.8	0.9	0.9	1.0	0.9	0.9
n		29	29	29	29	29	29	29	29	29	29	29	29	29	29	29	29
		p=0.31	p=0.57	p=0.39	p=0.07	p=0.02	p=0.41	p=0.21	p=0.60	p=0.56	p=0.83	p=0.52	p=0.40	p=0.31	p=0.69	p=0.53	p=0.21

Table S10. Immittance data for F-2 flight line participants

Subject		SC	Peak P	ECV	AR500-I	AR1000-I	AR2000-I	AR500-C	AR1000-C	AR2000-C
**Control**	**Mean**	**0.58**	**-5.21**	**1.29**	**86.07**	**87.32**	**88.33**	**95.19**	**93.96**	**94.07**
**Right Ear**	**STD**	0.26	12.81	0.33	6.3	5.7	5.9	5.6	4.7	5.4
all Iruma + HY	**SEM**	**0.04**	**2.20**	**0.06**	**1.2**	**1.1**	**1.1**	**1.1**	**0.9**	**1.0**
Yokota	**n**	**34**	**34**	**34**	**28**	**28**	**30**	**26**	**27**	**29**
**F-2 Right**	**Mean**	**0.57**	**-1.14**	**1.25**	**86.43**	**86.67**	**91.43**	**91.00**	**93.00**	**94.17**
**Ear**	**STD**	0.20	3.08	0.17	6.90	7.53	8.52	7.42	2.74	3.76
	**SEM**	**0.077**	**1.16**	**0.066**	**2.61**	**2.85**	**3.22**	**3.32**	**1.22**	**1.54**
	**n**	**7**	**7**	**7**	**7**	**7**	**7**	**5**	**5**	**6**
		p=0.92	p=0.41	p=0.76	p=0.90	p=0.80	p=0.26	**p=0.16**	p=0.66	p=0.97
**Conrol**	**Mean**	**0.67**	**-8.00**	**1.27**	**85.5**	**87.0**	**88.9**	**92.4**	**93.8**	**92.7**
**Left Ear**	**STD**	0.30	12.12	0.32	5.4	5.2	5.9	6.6	5.9	5.9
all Iruma + HY	**SEM**	**0.05**	**2.08**	**0.06**	**1.0**	**0.9**	**1.0**	**1.4**	**1.2**	**1.2**
Yokota	**n**	34	34	34	32	32	32	21	24	24
**F-2 Left**	**Mean**	**0.61**	**-2.57**	**1.26**	**83.33**	**82.50**	**87.86**	**91.67**	**94.00**	**96.00**
**Ear**	**STD**	0.22	4.50	0.17	5.16	4.18	6.99	7.64	8.94	4.18
	**SEM**	**0.082**	**1.70**	**0.065**	**1.95**	**1.58**	**2.64**	**4.41**	**4.00**	**1.87**
	**n**	**7**	**7**	**7**	**7**	**7**	**7**	**3**	**5**	**5**
		p=0.62	p=0.25	p=0.94	p=0.34	**p=0.039**	p=0.68	p=0.86	p=0.95	p=0.25

SC=static compliance (cc); Peak P=peak pressure (daPa); ECV=equivalent ear canal volume (cc); AR=acoustic reflex (dB); C=contralateral

Table S11. ABR results for F-2 flight line participants with values in milliseconds

Subject		Wave I Latency	Wave V Latency	I-III Latency	III-V Latency	I-V Latency	Wave I Amp	Wave V Amp	Wave V /Wave 1 amps	amplitude /latency* Wave 1	amplitude /latency Wave V
**Control**	**Mean**	**1.67**	**5.68**	**2.21**	**1.84**	**4.03**	**0.24**	**0.45**	**2.55**	**0.15**	**0.080**
**Right Ear Slow**	**STD**	0.21	0.24	0.27	0.12	0.20	0.14	0.16	1.62	0.094	0.030
all Iruma +	**SEM**	**0.036**	**0.041**	**0.046**	**0.021**	**0.035**	**0.023**	**0.027**	**0.28**	**0.016**	**0.005**
HY + Yokota	**n**	34	34	34	34	34	34	34	34	34	34
**F-2 Right**	**Mean**	**1.65**	**5.65**	**2.10**	**1.89**	**3.99**	**0.21**	**0.49**	**2.48**	**0.13**	**0.09**
**Ear Slow**	**STD**	0.095	0.154	0.148	0.068	0.142	0.060	0.13	0.95	0.041	0.024
	**SEM**	**0.036**	**0.058**	**0.056**	**0.026**	**0.054**	**0.023**	**0.049**	**0.36**	**0.016**	**0.009**
	**n**	7	7	7	7	7	7	7	7	7	7
		p=0.81	p=0.75	p=0.31	p=0.30	p=0.62	p=0.58	p=0.54	p=0.91	p=0.59	p=0.41
**Control**	**Mean**	**1.58**	**5.70**	**2.22**	**1.87**	**4.09**	**0.26**	**0.43**	**2.13**	**0.17**	**0.08**
**Left Ear Slow**	**STD**	0.10	0.21	0.18	0.15	0.23	0.11	0.13	1.51	0.073	0.024
all Iruma +	**SEM**	**0.018**	**0.035**	**0.031**	**0.026**	**0.039**	**0.019**	**0.022**	**0.26**	**0.013**	**0.004**
HY + Yokota	**n**	34	34	34	34	34	34	34	34	34	34
**F-2 Left**	**Mean**	**1.62**	**5.67**	**2.15**	**1.90**	**4.06**	**0.21**	**0.49**	**2.56**	**0.13**	**0.087**
**Ear Slow**	**STD**	0.055	0.16	0.067	0.13	0.12	0.081	0.17	0.89	0.052	0.033
	**SEM**	**0.021**	**0.062**	**0.025**	**0.048**	**0.046**	**0.031**	**0.066**	**0.34**	**0.020**	**0.013**
	**n**	7	7	7	7	7	7	7	7	7	7
		p=0.31	p=0.72	p=0.32	p=0.63	p=0.74	p=0.26	p=0.30	p=0.47	**p=0.18**	p=0.51

*amplitude/latency ratios (µV/ms, termed magnitude responses)

Table S12. Immittance data for Hyakuri AB F-4 flight line participants

Subject		SC	Peak P	ECV	AR500-I	AR1000-I	AR2000-I	AR500-C	AR1000-C	AR2000-C
**Control**	**Mean**	**0.58**	**-5.21**	**1.29**	**86.07**	**87.32**	**88.33**	**95.19**	**93.96**	**94.07**
**Right Ear**	**STD**	0.26	12.81	0.33	6.3	5.7	5.9	5.6	4.7	5.4
all Iruma + HY +	**SEM**	**0.04**	**2.20**	**0.06**	**1.2**	**1.1**	**1.1**	**1.1**	**0.9**	**1.0**
Yokota	**n**	**34**	**34**	**34**	**28**	**28**	**30**	**26**	**27**	**29**
**F-4**	**Mean**	**0.66**	**-3.47**	**1.34**	**86.79**	**89.29**	**91.00**	**93.93**	**93.89**	**95.77**
**Right Ear**	**STD**	0.33	11.23	0.49	5.04	5.14	6.04	8.13	5.46	4.94
	**SEM**	**0.09**	**2.90**	**0.12**	**1.35**	**1.37**	**1.56**	**2.17**	**1.82**	**1.37**
	**n**	15	15	16	14	14	15	14	9	13
		p=0.37	p=0.65	p=0.67	p=0.71	p=0.28	**p=0.16**	p=0.57	p=0.97	p=0.34
**Conrol**	**Mean**	**0.67**	**-8.00**	**1.27**	**85.5**	**87.0**	**88.9**	**92.4**	**93.8**	**92.7**
**Left Ear**	**STD**	0.30	12.12	0.32	5.4	5.2	5.9	6.6	5.9	5.9
all Iruma + HY +	**SEM**	**0.05**	**2.08**	**0.06**	**1.0**	**0.9**	**1.0**	**1.4**	**1.2**	**1.2**
Yokota	**n**	34	34	34	32	32	32	21	24	24
**F-4**	**Mean**	**0.73**	**-7.00**	**1.30**	**86.33**	**87.67**	**90.31**	**91.11**	**95.00**	**93.57**
**Left Ear**	**STD**	0.59	15.24	0.43	5.16	6.51	6.94	6.01	4.47	5.69
	**SEM**	**0.15**	**3.94**	**0.11**	**1.33**	**1.68**	**1.74**	**2.00**	**1.35**	**1.52**
	**n**	15	15	16	15	15	16	9	11	14
		p=0.64	p=0.81	p=0.78	p=0.62	p=0.71	p=0.47	p=0.62	p=0.55	p=0.66

SC=static compliance (cc); Peak P=peak pressure (daPa); ECV=equivalent ear canal volume (cc); AR=acoustic reflex (dB); C=contralateral

Table S13. DPOAE data for Hyakuri AB F-4 flight line participants with values in Hz

Subject		2000	2198	2416	2656	2920	3210	3529	3880	4265	4688	5154	5666	6229	6847	7527	8275
**Control**	**Mean**	**20.7**	**21.2**	**20.5**	**22.6**	**22.8**	**23.6**	**25.3**	**25.9**	**27.6**	**27.4**	**29.6**	**30.3**	**28.3**	**29.0**	**22.5**	**22.7**
**Right Ear**	**STD**	10.0	6.9	5.5	5.8	5.7	5.9	6.3	6.6	5.7	6.0	7.0	7.6	8.9	8.4	7.3	7.1
all Iruma +	**SEM**	**1.77**	**1.21**	**0.98**	**1.03**	**1.02**	**1.05**	**1.11**	**1.16**	**1.01**	**1.06**	**1.23**	**1.35**	**1.58**	**1.48**	**1.29**	**1.25**
HY + Yokota	**n**	32	32	32	32	32	32	32	32	32	32	32	32	32	32	32	32
**F-4**	**Mean**	**20.75**	**21.24**	**20.79**	**20.86**	**22.95**	**24.17**	**25.24**	**26.55**	**27.15**	**28.60**	**29.31**	**30.05**	**29.64**	**29.06**	**22.58**	**21.52**
**Right Ear**	**STD**	4.13	4.57	5.14	4.81	3.60	4.06	3.62	4.11	4.02	4.03	5.64	6.49	5.41	6.87	6.05	6.18
	**SEM**	**1.00**	**1.11**	**1.25**	**1.17**	**0.87**	**0.98**	**0.88**	**1.00**	**0.98**	**0.98**	**1.37**	**1.57**	**1.31**	**1.67**	**1.47**	**1.50**
	**n**	17	17	17	17	17	17	17	17	17	17	17	17	17	17	17	17
		p=0.98	p=0.98	p=0.86	p=0.30	p=0.92	p=0.72	p=0.97	p=0.71	p=0.77	p=0.46	p=0.88	p=0.91	p=0.57	p=0.98	p=0.97	p=0.57
**Conrol**	**Mean**	**21.61**	**20.70**	**20.79**	**22.35**	**23.31**	**23.01**	**25.06**	**26.72**	**27.41**	**27.94**	**28.98**	**30.31**	**29.49**	**28.58**	**22.86**	**21.01**
**Left Ear**	**STD**	5.85	5.28	5.65	6.08	5.93	6.85	5.82	5.89	5.81	6.27	7.05	6.87	6.90	7.76	7.24	7.12
all Iruma +	**SEM**	**1.03**	**0.93**	**1.00**	**1.08**	**1.05**	**1.21**	**1.03**	**1.04**	**1.03**	**1.11**	**1.25**	**1.21**	**1.22**	**1.37**	**1.28**	**1.26**
HY + Yokota	**n**	32	32	32	32	32	32	32	32	32	32	32	32	32	32	32	32
**F-4**	**Mean**	**20.94**	**19.01**	**19.56**	**21.02**	**20.29**	**22.28**	**22.49**	**25.93**	**26.61**	**27.22**	**28.77**	**30.26**	**28.77**	**27.74**	**23.46**	**23.24**
**Left Ear**	**STD**	4.48	5.06	4.54	4.53	5.27	5.45	6.30	5.36	4.91	4.85	5.39	5.64	5.97	6.75	6.24	5.70
	**SEM**	**1.09**	**1.23**	**1.10**	**1.10**	**1.28**	**1.32**	**1.53**	**1.30**	**1.19**	**1.18**	**1.31**	**1.37**	**1.45**	**1.64**	**1.51**	**1.38**
	**n**	17	17	17	17	17	17	17	17	17	17	17	17	17	17	17	17
		p=0.68	p=0.28	p=0.44	p=0.43	**p=0.085**	p=0.71	**p=0.16**	p=0.65	p=0.63	p=0.68	p=0.92	p=0.98	p=0.72	p=0.71	p=0.77	p=0.27

Table S14. Audiogram data for F-16 flight line participants measured in decibels Hearing Level (dB HL)

Subject		500 Hz	1000 Hz	2000 Hz	3000 Hz	4000 Hz	6000 Hz
Control	Mean	8.33	8.00	5.00	7.33	9.33	2.67
Right Ear	STD	6.45	4.14	5.98	7.29	8.63	9.04
	SEM	1.67	1.07	1.54	1.88	2.23	2.33
	n	15	15	15	15	15	15
Exposed	Mean	8.89	10.00	5.28	8.06	5.83	6.11
Right Ear	STD	3.23	3.43	4.36	3.89	6.00	7.58
	SEM	0.76	0.81	1.03	0.92	1.41	1.79
	n	18	18	18	18	18	18
		p=0.75	**p=0.14**	p=0.88	p=0.72	**p=0.18**	p=0.24
Control	Mean	5.67	5.33	7.00	7.33	9.33	4.33
Left Ear	STD	4.17	4.81	6.49	7.53	8.63	6.51
	SEM	1.08	1.24	1.68	1.94	2.23	1.68
	n	15	15	15	15	15	15
Exposed	Mean	7.50	8.61	5.56	9.72	7.22	4.44
Left Ear	STD	6.24	5.89	6.16	7.17	7.52	9.38
	SEM	1.47	1.39	1.45	1.69	1.77	2.21
	n	18	18	18	18	18	18
		p=0.34	**p=0.094**	p=0.52	p=0.36	p=0.46	p=0.97

Table S15. DPOAE data for Misawa AB F-16 flight line participants with values in Hz

Subject		2000	2198	2416	2656	2920	3210	3529	3880	4265	4688	5154	5666	6229	6847	7527	8275
Control	Mean	23.57	23.22	23.15	24.57	25.45	25.95	27.33	27.55	26.72	28.55	30.88	31.77	29.00	28.80	21.22	18.52
Right Ear	STD	4.15	4.83	3.55	3.14	3.66	4.09	3.68	5.37	5.67	5.06	3.44	4.84	6.30	7.58	5.28	6.92
	SEM	1.07	1.25	0.92	0.81	0.94	1.06	0.95	1.39	1.46	1.31	0.89	1.25	1.63	1.96	1.36	1.79
	n	15	15	15	15	15	15	15	15	15	15	15	15	15	15	15	15
Exposed	Mean	23.76	23.71	23.32	24.57	25.06	25.25	26.93	25.87	25.29	27.41	29.90	29.78	28.85	28.26	20.83	20.94
Right Ear	STD	7.13	8.19	7.77	7.07	6.24	5.97	6.57	5.94	6.24	4.49	6.88	6.48	7.31	6.46	3.87	5.10
	SEM	1.68	1.93	1.83	1.67	1.47	1.41	1.55	1.40	1.47	1.06	1.62	1.53	1.72	1.52	0.91	1.20
	n	18	18	18	18	18	18	18	18	18	18	18	18	18	18	18	18
		p=0.93	p=0.84	p=0.94	p=1.0	p=0.83	p=0.70	p=0.84	p=0.40	p=0.50	p=0.50	p=0.62	p=0.33	p=0.95	p=0.83	p=0.81	p=0.26
Control	Mean	21.35	23.26	22.57	23.28	22.81	24.40	26.64	27.49	26.55	27.95	30.63	31.52	30.72	29.80	21.69	19.87
Left Ear	STD	5.48	3.45	3.31	4.13	3.59	4.47	5.20	5.22	8.06	6.96	6.79	8.78	6.56	7.14	6.60	5.42
	SEM	1.41	0.89	0.86	1.07	0.93	1.15	1.34	1.35	2.08	1.80	1.75	2.27	1.69	1.84	1.70	1.40
	n	15	15	15	15	15	15	15	15	15	15	15	15	15	15	15	15
Exposed	Mean	23.00	23.18	22.54	23.54	23.58	25.57	26.24	26.77	25.99	27.79	30.05	30.85	29.37	26.71	21.02	19.42
Left Ear	STD	7.48	7.48	6.11	5.97	5.84	5.91	5.36	5.82	7.34	6.26	5.59	5.47	5.10	5.80	3.95	4.45
	SEM	1.76	1.76	1.44	1.41	1.38	1.39	1.26	1.37	1.73	1.48	1.32	1.29	1.20	1.37	0.93	1.05
	n	18	18	18	18	18	18	18	18	18	18	18	18	18	18	18	18
		p=0.48	p=0.97	p=0.99	p=0.89	p=0.66	p=0.53	p=0.83	p=0.71	p=0.84	p=0.95	p=0.79	p=0.79	p=0.51	**p=0.18**	p=0.72	p=0.80

Figure S1. Comparison of the total VOCs in blood, urine prior to shift and urine post shift in smokers and non-smokers

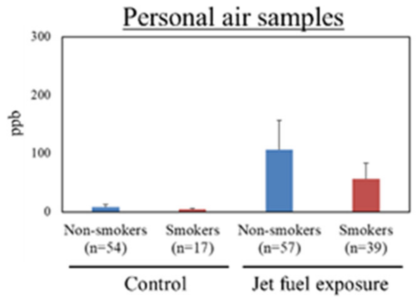

	NC	SC	NE	SE
NC		n,.s.	NE >	SE >
SC	n,.s.		NE >	SE >
NE	NE >	NE >		n,.s.
SE	SE >	SE >	n,.s.	

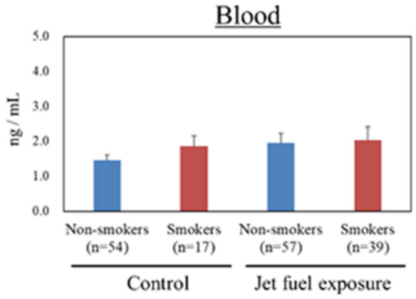

	NC	SC	NE	SE
NC		n,.s.	n,.s.	n,.s.
SC	n,.s.		n,.s.	n,.s.
NE	n,.s.	n,.s.		n,.s.
SE	n,.s.	n,.s.	n,.s.	

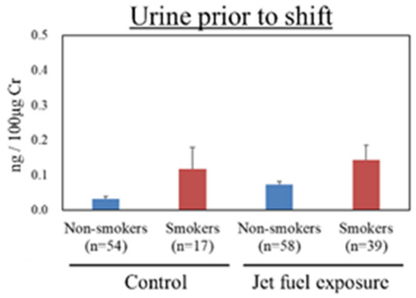

	NC	SC	NE	SE
NC		n,.s.	NE >	SE >
SC	n,.s.		n,.s.	n,.s.
NE	NE >	n,.s.		n,.s.
SE	SE >	n,.s.	n,.s.	

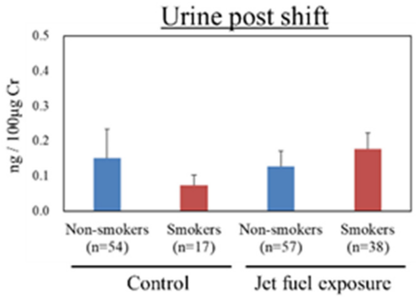

	NC	SC	NE	SE
NC		n,.s.	NE >	SE >
SC	n,.s.		n,.s.	n,.s.
NE	NE >	n,.s.		n,.s.
SE	SE >	n,.s.	n,.s.	
